# Epithelial AhR Suppresses Allergen-Induced Oxidative Stress and Senescence via c-Myc Regulation

**DOI:** 10.3390/antiox15010022

**Published:** 2025-12-23

**Authors:** Zhifeng Chen, Wenjing Gu, Rongjun Wan, Yixiang Zeng, Xudong Xiang, Ruoyun Ouyang, Peisong Gao

**Affiliations:** 1 Division of Allergy and Clinical Immunology, Johns Hopkins University School of Medicine, Baltimore, MD 21205, USAwgu16@jh.edu (W.G.);; 2 Department of Pulmonary and Critical Care Medicine, The Second Xiangya Hospital, Central South University, Changsha 410011, China; 3 Department of Respiratory Medicine, Children’s Hospital of Soochow University, Suzhou 215025, China; 4 Department of Pulmonary and Critical Care Medicine, The First Affiliated Hospital of Wannan Medical College, Wuhu 241001, China; 5 The Johns Hopkins Asthma & Allergy Center, 5501 Hopkins Bayview Circle, Baltimore, MD 21224, USA

**Keywords:** allergen, asthma, ROS, aryl hydrocarbon receptor, c-Myc, cellular senescence, airway epithelial cells

## Abstract

Environmental allergens trigger epithelial reactive oxygen species (ROS) production and cellular senescence, contributing to airway inflammation. The aryl hydrocarbon receptor (AhR), a ligand-activated transcription factor responsive to environmental stimuli, may modulate this process. Single-cell transcriptomics from allergen-challenged bronchoalveolar brushings of allergic asthma and non-asthmatic allergic control subjects were analyzed for ROS, senescence, and AhR activity. Club cell-specific p16 knockout (*p16^ΔScgb1a1^*) and AhR-deficient (*AhR^ΔScgb1a1^*) mice were used to assess epithelial senescence and AhR function. Single-cell analysis revealed epithelial senescence as a hallmark of allergen-induced asthma. *p16^ΔScgb1a1^* mice exhibited reduced ROS levels and airway inflammation. Single-cell analysis also demonstrated increased AhR activity and ROS generation in airway epithelial cells of allergen-treated asthmatics, and ROS correlated positively with AhR activity and senescence. It was documented that the regulation of AhR on senescence was attenuated by VAF347, whereas AhR deficiency exacerbated ROS generation and inflammation in *AhR^ΔScgb1a1^* mice. RNA-seq identified senescence as a key AhR-regulated pathway, implicating c-Myc, TGF-β2, and SERPINE1 as major targets. AhR binding to the c-Myc promoter was confirmed by ChIP-PCR, and pharmacologic inhibition of c-Myc with EN4 reduced allergen-induced ROS, senescence, and inflammation. These findings demonstrate that epithelial AhR suppresses allergen-induced ROS generation and cellular senescence via direct regulation of c-Myc.

## 1. Introduction

The prevalence of asthma has continued to rise despite increased awareness of its hazards and substantial efforts in prevention and treatment [1]. Notably, elderly patients (≥65 years) experience a significantly higher incidence of acute exacerbations compared with younger patients [2,3,4]. They also tend to exhibit more severe airway obstruction, poorer asthma control, and more frequent hospitalizations. Recently, increasing attention has been focused on the potential involvement of cellular senescence in asthma. Although data remain limited, emerging evidence suggests that cellular senescence is accelerated in asthma, particularly in elderly patients [5,6,7,8,9]. Cellular senescence is a complex state induced by factors such as cellular stress and DNA damage, leading to cell cycle arrest and the release of senescence-associated secretory phenotype (SASP) factors [10,11,12]. The SASP includes chemokines, cytokines, growth factors, adhesion molecules, and lipids, which exert both local and systemic effects that contribute to disease pathogenesis [13,14,15,16,17,18,19,20]. Targeting senescence has therefore emerged as a promising therapeutic approach for preventing or treating disease [21]. However, the specific pathways driving cellular senescence and the potential of senescence-targeted therapies in asthma remain poorly understood.

Increasing evidence suggests that airway epithelial cells in asthma, particularly in elderly patients, may undergo accelerated cellular senescence [5,6,7,8,9]. Upon contact with aeroallergens such as cockroach, house dust mite, or pollen, epithelial cells generate excessive reactive oxygen species (ROS) through NADPH oxidase activation and mitochondrial dysfunction, leading to oxidative DNA damage and activation of p53-p21 and p16-RB senescence pathways [22,23,24]. Importantly, allergen-induced oxidative stress impairs autophagic flux, causing damaged mitochondria and proteins to accumulate, which amplifies ROS and SASP release [25], thereby driving epithelial barrier dysfunction, alarmin secretion (e.g., TSLP, IL-33, and IL-25), and allergic airway inflammation [8,22].

Aryl hydrocarbon receptor (AhR), a cytoplasmic receptor and a ligand-activated transcription factor, has been suggested to regulated asthma phenotypes in elderly populations as part of the aging process [4,26]. Our research has established the AhR as a critical protective regulator in airway epithelial cells during allergic airway inflammation. Specifically, AhR activation suppressed mucus hyperproduction by inhibiting ROS-triggered NLRP3 inflammasome activation [27]. Furthermore, we demonstrated that AhR in type II alveolar epithelial (AT2) cells preserves autophagic flux, preventing the accumulation of damaged organelles and limiting inflammatory responses [28]. These findings align with emerging data demonstrating that environmental pollutants such as PM_2.5_ and vehicular exhaust particles exacerbate airway hyperresponsiveness and inflammation via AhR-dependent pathways, including ROS production and AhR-Notch signaling [29,30]. These findings suggest that AhR modulates key stress response pathways in airway epithelial cells, oxidative stress, inflammasome activation, and autophagy, that may also be central drivers of allergen-induced cellular senescence.

In this study, we investigated the role of the AhR in regulating allergen-induced ROS generation and cellular senescence in bronchial epithelial cells and its contribution to allergic airway inflammation. Leveraging a publicly available human scRNA-seq dataset (GSE193816), we analyzed the expression of senescence, oxidative stress, and *AhR* activation signatures across airway epithelial and immune cell subsets. This dataset includes cells directly obtained from the lower airway via endobronchial brushings from allergic asthmatics and non-asthmatic allergic controls, with or without allergen challenge [31]. Using both *p16^ΔScgb1a1^* and *AhR^ΔScgb1a1^* mice along with human bronchial epithelial cells, we demonstrated that AhR is a key regulator of epithelial ROS generation and senescence during allergen exposure. RNA-seq analysis identified cellular senescence as a major AhR-regulated pathway, with c-Myc emerging as a central downstream target. These findings reveal that AhR modulates allergen-induced senescence via a c-Myc-dependent mechanism.

## 2. Methods

### 2.1. Single-Cell RNA-Seq Data Acquisition and Pre-Processing

Raw count matrices for human asthma models were retrieved from the Gene Expression Omnibus (accession GSE193816). The study provides pre-annotated single-cell transcriptomes from broncho-alveolar brushings collected at baseline, after diluent (solution) challenge and after allergen challenge in patients with allergic asthma (AA, *n* = 4) and non-asthmatic allergic controls (AC, *n* = 4) [31]. Detailed demographic and clinical characteristics of the participating subjects were presented in Table S1. The allergen-challenged cells (AA_Ag and AC_Ag) and baseline (AA_Pre and AC_Pre) were retained for downstream analysis. Analyses were performed using Seurat (5.3.0), gene counts were log-normalized, and the top 2000 highly variable genes were identified with the vst method. Data was scaled to unit variance and zero mean before dimensionality reduction. The harmony algorithm was used to remove the batch effect caused by sequencing channels.

### 2.2. Mice

Both *p16^ΔScgb1a1^* and *AhR^ΔScgb1a1^* mice on a C57BL/6 background were generated by crossing *CC10-Cre^ERTM^* mice with *Cdkn2a^f/f^* or *AhR^f/f^* mice, respectively. All parental strains were obtained from The Jackson Laboratory (Bar Harbor, ME, USA). Mice were maintained under specific pathogen-free conditions in the animal facilities of the Johns Hopkins University School of Medicine. All procedures were performed in accordance with institutional and National Institutes of Health guidelines and were approved by the Johns Hopkins University Animal Care and Use Committee. Experimental mice were used at ≥8 weeks of age, with age- and sex-matched littermates serving as controls.

### 2.3. Cockroach Allergen-Induced Asthma Mouse Model

Before induction of allergic asthma, *p16^ΔScgb1a1^* and *AhR^ΔScgb1a1^* mice received tamoxifen (75 mg/kg; Sigma, Rockville, MD, USA) via intraperitoneal injection for 5 consecutive days, followed by a 1-week rest period, in accordance with the Jackson Laboratory protocol. Allergic airway inflammation was then induced using cockroach extract (CRE; Greer Laboratories, Lenoir, NC, USA), a well-established cockroach allergen–induced T2-high allergic airway inflammation model, in which eosinophils are the predominant inflammatory cell type in the BALF [32]. Briefly, under light isoflurane anesthesia, mice were intratracheally instilled with 20 μg CRE (B46, Greer Laboratories) in 50 μL phosphate-buffered saline (PBS) for sensitization and subsequently challenged with the same dose of CRE. Control mice received PBS alone. Mice were euthanized one day after the final challenge for collection of lung tissue, bronchoalveolar lavage fluid (BALF), and blood. BALF was obtained by flushing the lungs twice with 1.0 mL ice-cold PBS. Blood samples were centrifuged to obtain serum for further analysis. In selected experiments, mice were treated with the AhR agonist VAF347 (30 mg/kg; MCE, Aschersleben, Germany) or the c-Myc inhibitor EN4 (50 mg/kg; MCE), administered by gavage or intraperitoneal injection 1 h prior to each allergen challenge [33,34]; control mice received saline vehicle.

### 2.4. Analysis of Lung Inflammation

The mouse lungs were perfused by injecting 10 mL of ice-cold PBS into the right ventricle. Subsequently, the left lung was excised, fixed with 4% formalin, and then embedded in paraffin or optimal cutting temperature (OCT) compound. Sections (4 μm) were prepared from these paraffin-embedded lungs and stained with hematoxylin and eosin (H&E) as well as periodic acid-Schiff (PAS) to assess pulmonary pathology and mucus secretion. The right lung was frozen at −80 °C for further detection.

### 2.5. Flow Cytometry Analysis

The BALFs obtained in the previous step were centrifuged, and the supernatant was collected for later use. The cell pellet was resuspended with pre-cooled PBS to prepare a cell suspension. The total number of cells in the BALFs was counted using Countless II (ThermoFisher, Waltham, MA, USA). The differential cell percentages in the BALFs were measured on an Accuri C6 Plus flow cytometer (BD Biosystems, Milpitas, CA, USA), and the data were analyzed using FlowJo software(version 10.8.1) (Tree Star Inc., Ashland, OR, USA) [35]. The antibodies were provided in the online repository (Supplementary Table S1).

### 2.6. Immunofluorescence Staining

Immunofluorescence staining was performed as previously reported [36]. Briefly, first, lung tissue sections were blocked with 5% *w*/*v* bovine serum albumin (BSA) at room temperature for 1 h. For cell samples, they were first fixed with 4% paraformaldehyde for half an hour and then blocked. Then, the samples were incubated with the primary antibodies listed in the online repository (Supplementary Table S2) at 4 °C overnight. Subsequently, the samples were incubated with fluorescent-conjugated secondary antibodies at room temperature for 1 h. Isotype-matched negative control antibodies were used under the same conditions. The sections were mounted with a fluorescent mounting medium (Sigma) containing DAPI (4′,6-diamidino-2-phenylindole) (ThermoFisher) and then observed using a NIKON ECLIPSE Ti-U microscope (Nikon, Melville, NY, USA) equipped with a DS-Fi2 camera.

### 2.7. ROS Measurement

For ROS in lung tissues, frozen sections were incubated with 5 µM of dihydroethidium (DHE, Thermo Fisher Scientific) 37 °C for 30 min, and then fluorescent signal was observed and analyzed under microscope (Nikon, Chiyoda, Japan).

### 2.8. Senescence-Associated β-Galactosidase Staining

According to the manufacturer’s instructions, the cultured cells and frozen lung tissue sections (10 µm) were fixed and stained using the SA-β-Gal staining kit (9860S, Cell Signaling Technology, Danvers, MA, USA).

### 2.9. Enzyme-Linked Immunosorbent Assay

Interleukin-4 (IL-4), IL-5, IL-13, IL-17, and IFN-γ in the cell-free BALF were detected using enzyme-linked immunosorbent assay (ELISA) kits according to the manufacturer’s instructions [37].

### 2.10. Cell Culture and Treatment

Human bronchial epithelial cells (HBECs, Sigma-Aldrich), a well-established immortalized cell line, were maintained in Dulbecco’s Modified Eagle Medium supplemented with 10% *v*/*v* fetal bovine serum and 1% penicillin-streptoc-Mycin in a humidified atmosphere at 37 °C and 5% CO_2_. HBECs were treated with cockroach extract (CRE, Greer Laboratory) alone, or were pre-treated with the AhR agonist VAF347 (HY-135750, 20 μM, MCE), the AhR antagonist CH-223191 (C8124, 10 μM, Sigma), or the c-Myc inhibitor EN4 (HY-134761, 50 μM, MCE) 1 h before CRE treatment [28,33,38].

### 2.11. RNA-Seq Analysis

According to the manufacturer’s instructions, the total RNA of each sample was processed using the Vazyme VAHTS Universal V10 RNA-seq Library Prep Kit (Nanjing, China), which selectively captures mRNA transcripts through poly(A) enrichment. Subsequently, the enriched RNA was reverse-transcribed and constructed into a sequencing library following the manufacturer’s protocol. The library was sequenced using paired-end 2 × 150 bp sequencing on the Element AVITI system, and approximately 20 million paired-end reads were obtained for each sample. Gene expression levels were defined as Salmon counts. Differential gene expression analysis was performed using the R package DESeq2 (version 1.46.0). Genes with an absolute log_2_ fold change [abs(log_2_FC)] > 0.5 and *p* < 0.05 were defined as differentially expressed genes (DEGs). Gene set enrichment analysis was carried out using clusterProfiler version 4.14.4 [39]. Upregulated or downregulated DEGs were visualized using ComplexHeatmap (version 2.22.0) and ggplot2 (version 3.5.1) [40]. All downstream analyses and visualizations based on RNA-seq were conducted in R version 4.4.2.

### 2.12. RNA Isolation and Quantitative Real-Time PCR Analysis

Total RNA was extracted from lung tissues or HBECs using the Monarch Total RNA Miniprep Kit (New England Biolabs, Ipswich, MA, USA), and cDNA was synthesized using the High-Capacity cDNA Reverse Transcription Kit (Thermo Fisher). Quantitative real-time PCR analysis was performed on an ABI Prism 7300 Detection System using Power SYBR Green PCR Master Mix (Thermo Fisher). Data were analyzed using the 2^−ΔΔCT^ method relative to the housekeeping genes β-actin or GAPDH. The primer sequences are provided in the online repository (Supplementary Table S3).

### 2.13. In Silico Prediction of AhR–c-Myc Binding Using AlphaFold 3

To predict the regulatory relationship between AhR and c-Myc, we obtained the protein sequence data from UniProtKB (https://www.uniprot.org/, accessed on 15 May 2025), with the UniProtKB IDs of P35869 for AhR and P01106 for c-Myc. Subsequently, we conducted molecular docking using the AlphaFold 3 Server platform. Finally, we visualized the results using PyMOL (Version 3.0.3).

### 2.14. Chromatin Immunocoprecipitation

To investigate the impact of AhR on the promoter region of c-Myc, a ChIP kit (Abcam, Cambridge, MA, USA) was employed, and the experiments were conducted following the manufacturer’s instructions. Briefly, after intervention, HBEs were cross-linked with 1% formaldehyde to stabilize protein–DNA interactions. Subsequently, the nuclei were isolated by centrifugation and lysis, and the DNA was fragmented by sonication. Then, the DNA-protein complexes were immunoprecipitated by incubating overnight at 4 °C with antibodies against AhR or rabbit IgG. After that, the DNA was eluted and purified. Finally, the obtained DNA was analyzed by qPCR. The primer sequences for c-Myc are provided in the online repository (Supplementary Table S4).

### 2.15. Statistical Analysis

All statistical analyses were conducted using GraphPad Prism version 10.1.2 (GraphPad Software, La Jolla, CA, USA). All data are presented as the mean ± SEM. For comparisons among more than two groups, an ordinary one-way analysis of variance (ANOVA) was performed, followed by Tukey’s post hoc test. A *p* value < 0.05 was considered to indicate a statistically significant difference.

## 3. Results

### 3.1. Single-Cell Transcriptomics Reveal Epithelial Senescence as a Key Feature of Allergen-Induced Asthma

**Figure 1:**
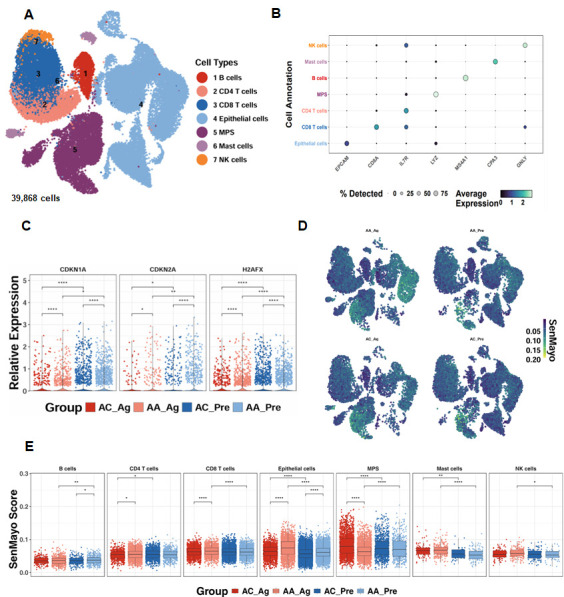
Single-cell transcriptomics reveal epithelial senescence as a key feature of allergen-induced asthma. (**A**) UMAP embedding of 39868single cells from allergic asthmatics (AA, *n* = 4) and allergic controls (AC, *n* = 4), color-coded by lineage. (**B**) Dot plot of canonical marker genes validating lineage identity. (**C**) Expression of senescence-associated genes (CDKN1A, CDKN2A, H2AFX) in airway epithelial cells. (**D**) UMAP embedding showing distribution of SenMayo senescence scores across cell subsets before and after allergen challenge. (**E**) SenMayo senescence scores in allergen-treated asthmatics (AA_Ag), allergen-treated allergic controls (AC_Ag), pretreated asthmatics (AA_Pre) and allergic controls (AC_Pre). * *p* < 0.05, ** *p* < 0.01, **** *p *< 0.0001.

To investigate the relevance of cellular senescence in asthma, we analyzed scRNA-seq data from the GSE193816 dataset, which included 39,868 lung cells obtained via endobronchial brushing from allergic asthmatics (AA, *n* = 4) and allergic controls (AC, *n* = 4) [31]. Unsupervised clustering identified seven major cell lineages using the Louvain algorithm (Figure 1A) [41], and lineage identity was validated through marker gene expression analysis, as illustrated by a dot plot showing both the proportion of cells expressing canonical markers and their relative expression levels (Figure 1B). Senescence-associated genes, including *CDKN1A*, *CDKN2A*, and *H2AFX*, were significantly upregulated in airway epithelial cells from both allergen-treated (AA_Ag) and untreated allergic asthmatics (AA_Pre) compared with their respective allergic controls (AC_Ag and AC_Pre). Notably, expression of these genes was further increased in allergen-treated epithelial cells (AA_Ag and AC_Ag) relative to their untreated counterparts (AA_Pre and AC_Pre) (Figure 1C). UMAP embedding of SenMayo senescence scores demonstrated broad enrichment of senescence signatures across cell subsets following allergen challenge (Figure 1D). Quantitative comparison revealed significantly higher senescence scores in AA_Ag compared with AC_Ag across multiple immune cell types, including CD4^+^ T cells and CD8^+^ T cells, with airway epithelial cells exhibiting the strongest enrichment (Figure 1E). Senescence scores were especially markedly elevated in airway epithelial cells from both AA_Ag and untreated AA_Pre compared with their corresponding allergic controls (AC_Ag and AC_Pre). These scores were further increased in allergen-treated epithelial cells (AA_Ag and AC_Ag) relative to their untreated counterparts (AA_Pre and AC_Pre). Together, these results demonstrate that allergen exposure induces increased cellular senescence, with airway epithelial cells being the primary compartment affected.

### 3.2. Deletion of Senescent Club Cells Attenuates Allergen-Induced ROS and Airway Inflammation

**Figure 2:**
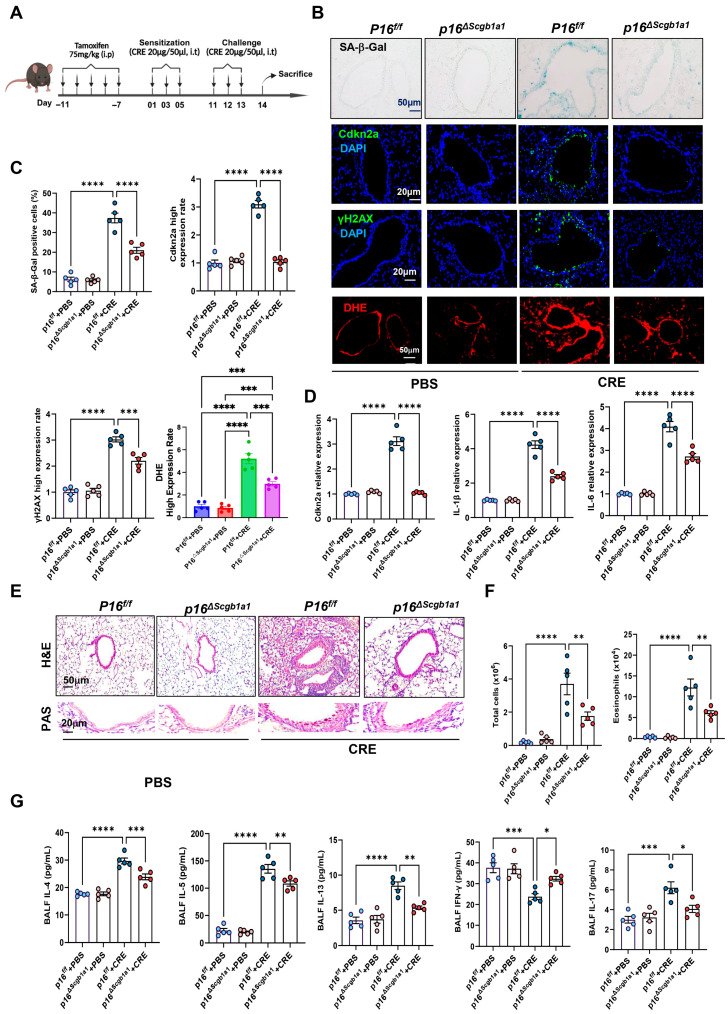
Deletion of senescent Club cells attenuates allergen-induced airway inflammation. (**A**) Experimental scheme of mouse asthma model. (**B**) Representative images of SA-β-Gal staining and Cdkn2a (p16), γH2Ax, and DHE immunostaining in lung tissue sections. (**C**) Quantitative analysis of relative fluorescence intensity for SA-β-Gal, Cdkn2a (p16), γH2Ax, and ROS. (**D**) RT-PCR analysis of Cdkn2a (p16), IL-6, and IL-1β in lung tissues. (**E**) Representative images of hematoxylin and eosin staining (H&E, upper) and periodic acid–Schiff staining (PAS, lower) of lung sections. (**F**) Total and eosinophil cell count in bronchoalveolar lavage fluid (BALF). (**G**) BALF cytokine levels measured by ELISA. *n* = 5. Data represent mean ± SEM; * *p* < 0.05, ** *p* < 0.01, *** *p* < 0.001, **** *p* < 0.0001.

To examine the functional significance of epithelial senescence in allergen-induced airway inflammation, we generated *p16^ΔScgb1a1^* mice by crossbreeding *p16^f/f^* with *Scgb1a1-CreER^TM^* mice (Figure S1A). Selective deletion of p16^+^ cells in Club cells was confirmed by genotyping (Figure S1B) and immunostaining (Figure S1C). These mice were then subjected to the CRE-induced asthma protocol (Figure 1A). In wild-type (WT) mice, CRE exposure markedly increased senescence markers, as demonstrated by SA-β-Gal staining and immunofluorescence for Cdkn2a (p16) and γH2AX. In contrast, *p16^ΔScgb1a1^* mice exhibited substantially reduced expression of these markers (Figure 1B,C). The same pattern was also observed for ROS generation. RT-PCR further validated decreased transcript levels of p16, IL-6, and IL-1β in lung tissues of *p16^ΔScgb1a1^* mice compared with WT controls (Figure 1D). Histological examination revealed attenuated peribronchial and perivascular inflammatory infiltrates (Figure 2E, upper) and reduced mucus hypersecretion in *p16^ΔScgb1a1^* mice (Figure 2E, lower). Consistently, bronchoalveolar lavage fluid (BALF) analysis showed significantly lower numbers of total inflammatory cells, with a marked reduction in eosinophils (Figure 2F and Figure S1D). Moreover, levels of cytokines IL-4, IL-5, IL-13, and IL-17 were significantly reduced in BALF from *p16^ΔScgb1a1^* mice compared with WT controls (Figure 2G). In contrast, CRE exposure suppressed the Th1 cytokine IFN-γ in WT mice; its levels were restored in *p16^ΔScgb1a1^* mice relative to WT controls. These findings demonstrate that selective deletion of senescent Club cells mitigates allergen-induced epithelial senescence, reduces mucus production, and alleviates allergic airway inflammation.

### 3.3. Allergen-Induced Upregulation of AhR Correlates with ROS Generation and Epithelial Senescence

**Figure 3:**
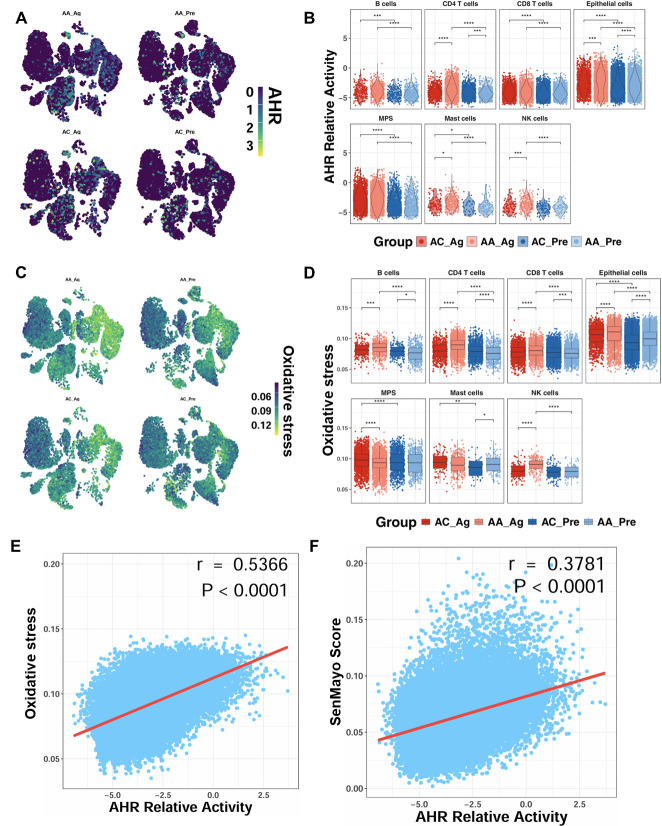
Enhanced AhR signaling in airway epithelial cells following allergen exposure. (**A**) UMAP embedding of single-cell data showing AhR expression across cell lineages before and after allergen challenge. (**B**) AhR activity scores in allergen-treated asthmatics (AA_Ag), allergen-treated allergic controls (AC_Ag), pretreated asthmatics (AA_Pre) and allergic controls (AC_Pre). (**C**,**D**) UMAP embedding single-cell data showing ROS expression across cell lineages following allergen challenge (**C**) and ROS scores in allergen-treated asthmatics (AA_Ag), allergen-treated allergic controls (AC_Ag), pretreated asthmatics (AA_Pre) and allergic controls (AC_Pre) (**D**). (**E**,**F**) Correlations between ROS levels and AhR activity (**E**) and between AhR activity and cellular senescence (**F**). Data represent mean ± SEM. * *p* < 0.05, ** *p* < 0.01, *** *p* < 0.001, **** *p* < 0.0001.

To determine whether allergen challenge alters AhR expression, we analyzed single-cell transcriptomic profiles from the GSE193816 dataset. UMAP embedding revealed broad AhR expression across multiple cell lineages (Figure 3A). Quantitative analysis showed significantly higher AhR activity scores in AA_Ag compared with AC_Ag across multiple cell types, including CD4^+^ T cells, epithelial cells, mast cells, and NK cells (Figure 3B). Notably, AhR activity scores were significantly higher in airway epithelial cells from both AA_Ag and untreated AA_Pre compared with their corresponding allergic controls (AC_Ag and AC_Pre). These scores were further increased in allergen-treated epithelial cells (AA_Ag and AC_Ag) relative to their untreated counterparts (AA_Pre and AC_Pre). Similarly, single-cell analysis further demonstrated a parallel pattern for ROS generation, showing increased ROS generation in allergen-treated asthmatics (Figure 3C,D), with the most pronounced elevation observed within epithelial cells. Notably, ROS levels correlated positively with AhR activity (*r* = 0.537, *p* < 0.0001; Figure 3E). AhR activity also correlated with cellular senescence (*r* = 0.378, *p* < 0.0001; Figure 3F). Thus, these findings indicate that allergen challenge promotes AhR activation in airway epithelial cells, which is closely associated with elevated ROS and senescence signatures.

### 3.4. Enhanced AhR Signaling Protects Against Allergen-Induced Senescence in HBECs

Given the enhanced AhR signaling observed in airway epithelial cells, we hypothesized that AhR signaling may serve as a central driver of allergen-induced cellular senescence. To test this, HBECs were exposed to CRE (50 μg/mL) for 24 h in the presence or absence of the AhR agonist VAF347 (20 μM) or antagonist CH223191 (10 μM). Expression of AhR and senescence-related markers was assessed by immunostaining (Figure S2A,B) and RT-PCR (Figure S2C). As expected, CRE increased SA-β-Gal activity in HBECs, which was further enhanced by CH223191 but suppressed by VAF347. Similar patterns were observed for immunofluorescent staining of Cdkn2a, Cdkn1a, and γ-H2AX. In addition, IL-1β and IL-6, representative SASP factors, were elevated after CRE exposure, augmented by CH223191, and reduced by VAF347 (Figure S2D). These in vitro findings indicate that AhR activation protects against allergen-induced cellular senescence.

### 3.5. Club Cell-Specific Deletion of AhR Exacerbates Cockroach Allergen-Induced Epithelial Senescence and Airway Inflammation

**Figure 4:**
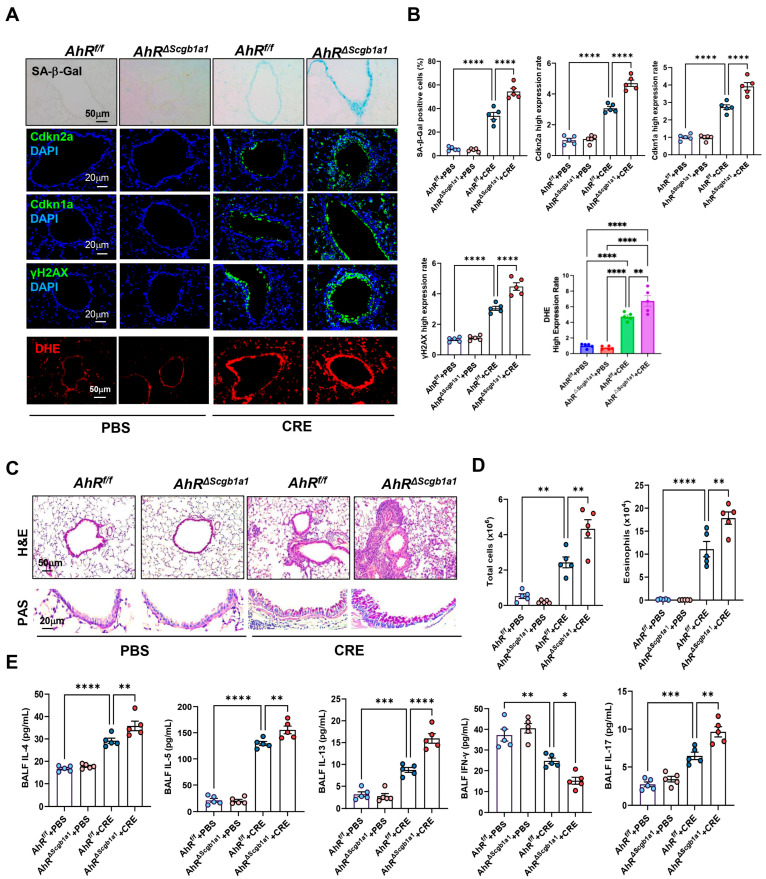
Club cell-specific deletion of AhR exacerbates cockroach allergen-induced epithelial senescence and airway inflammation. (**A**) Representative immunofluorescence staining for SA-β-Gal activity, senescence-associated markers, and ROS in lung sections. Nuclei were counterstained with DAPI. (**B**) Quantitative analysis of relative fluorescence intensity for SA-β-Gal, Cdkn2a (p16), γ-H2AX, and ROS from images in (**A**). (**C**) Representative images of hematoxylin and eosin staining (H&E, upper) and periodic acid-Schiff staining (PAS, lower) of lung sections. (**D**) Total and eosinophil cell count in bronchoalveolar lavage fluid (BALF). (**E**) BALF cytokine levels measured by ELISA. *n* = 5. Data represent mean ± SEM; * *p* < 0.05, ** *p* < 0.01, *** *p* < 0.001, **** *p* < 0.0001.

Next, we examined whether loss of AhR in Club cells would have the opposite effect. *AhR^ΔScgb1a1^* mice were generated by crossbreeding *AhR^f/f^* mice with *Scgb1a1-CreER^TM^* mice (Figure S3A), and efficient deletion of AhR in Club cells was confirmed by genotyping (Figure S3B) and immunostaining (Figure S3C). Following CRE exposure, as illustrated in Figure 2A, *AhR^ΔScgb1a1^* mice exhibited markedly increased SA-β-Gal activity and stronger immunofluorescent staining for senescence-associated markers compared with *AhR^f/f^* controls (Figure 4A). Quantitative fluorescence intensity analysis demonstrated significant upregulation of all three markers in AhR-deficient Club cells (Figure 4B). The same pattern was also observed for ROS generation. Consistent with these histologic observations, qRT-PCR analysis revealed elevated expression of Cdkn2a, and SASP-related cytokines (IL-1β, IL-6) in lung tissues from *AhR^ΔScgb1a1^* mice compared with controls (Figure S3D). Histopathologic evaluation showed more severe peribranchial and perivascular inflammatory infiltrates and increased mucus production in *AhR^ΔScgb1a1^* mice (Figure 4C). BALF analysis confirmed significantly higher total inflammatory cell counts, particularly eosinophils (Figure 4D and Figure S3E), along with increased levels of cytokines IL-4, IL-5, IL-13, and IL-17 in BALF from *p16^ΔScgb1a1^* mice compared with WT controls (Figure 4E). In contrast, CRE exposure suppressed the Th1 cytokine IFN-γ in WT mice; its levels were further reduced in *p16^ΔScgb1a1^* mice relative to WT controls. Collectively, these results suggest that pharmacologic activation of AhR protects against allergen-induced ROS, cellular senescence, and alleviates allergic airway inflammation.

### 3.6. AhR Activation Suppresses Cockroach Allergen-Induced Senescence and Airway Inflammation

To determine whether AhR activation modulates allergen-induced cellular senescence, we employed the CRE-induced asthma mouse model in the presence or absence of the AhR agonist VAF347 (Figure S4A). CRE exposure markedly increased SA-β-Gal activity and expression of senescence-associated markers [Cdkn2a (p16), Cdkn1a (p21), γ-H2AX] in the lung tissues, whereas VAF347 treatment significantly suppressed these responses (Figure S4B,C). qRT-PCR analysis confirmed that VAF347 reduced CRE-induced expression of Cdkn2a, IL-6, and IL-1β in lung tissues (Figure S4D). Histological analyses revealed that VAF347 attenuated peribronchial and perivascular inflammation and decreased mucus production (Figure S4E). Consistent with these histologic findings, VAF347 treatment significantly reduced total and differential inflammatory cell counts in BALF, particularly eosinophils (Figure S4F), and lowered BALF IL-4, IL-5, IL-13, and IL-17 but increased IFN-γ levels (Figure S4G). These results indicate that pharmacologic activation of AhR protects against allergen-induced cellular senescence and alleviates allergic airway inflammation.

### 3.7. Transcriptomic Profiling Identifies AhR-Regulated Gene Expression in HBECs

**Figure 5:**
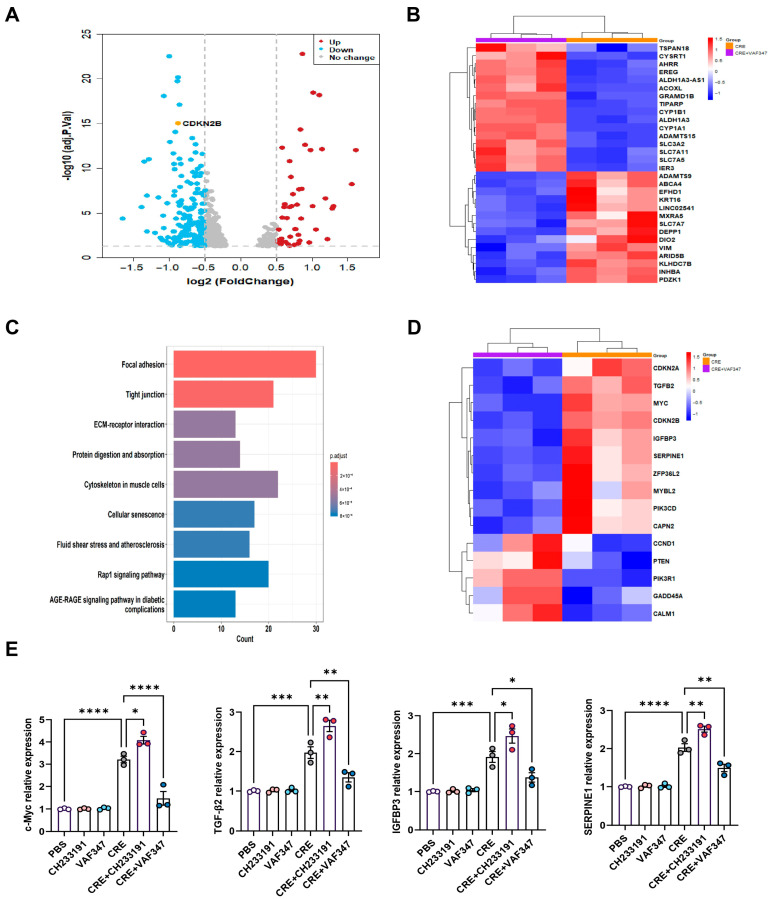
Transcriptomic profiling identifies AhR-regulated gene expression in HBECs. (**A**) Volcano plot of differentially expressed mRNAs in CRE-treated HBECs with or without the AhR agonist VAF347. (**B**) Heatmap of the top-ranking differentially expressed mRNAs in CRE-treated HBECs with or without VAF347. (**C**) KEGG pathway enrichment map of differentially expressed genes comparing the CRE and CRE + VAF347 groups. (**D**) Heatmap of differentially expressed genes enriched in the cellular senescence pathway. (**E**) Validation of selected senescence-related genes (*MYC*, *TGFβ2*, *IGFBP3*, and *SERPINE1*) by RT-PCR. *n* = 3. Data represent mean ± SEM; * *p* < 0.05, ** *p* < 0.01, *** *p* < 0.001, **** *p* < 0.0001.

To define the transcriptional programs regulated by AhR during allergen exposure, we performed RNA-seq on CRE-treated HBECs with or without the AhR agonist VAF347. Volcano plot analysis revealed a distinct transcriptional separation between the CRE and CRE + VAF347 groups (Figure 5A). A total of 585 mRNAs were differentially expressed in HBECs, including 566 down-regulated genes and 19 up-regulated genes. Heatmap visualization of the top differentially expressed genes confirmed robust activation of AhR signaling by VAF347, as evidenced by increased expression of canonical targets *AHRR*, *CYP1B1*, and *CYP1A1* (Figure 5B). KEGG pathway analysis identified cellular senescence as one of the most prominently altered pathways following AhR activation, along with focal adhesion, tight junctions, extracellular matrix-receptor interaction, and the Rap1 signaling pathway (Figure 5C). Within this pathway, key genes including *MYC*, *TGFβ2*, *IGFBP3*, and *SERPINE1* were markedly modulated by VAF347, as illustrated in the senescence-focused heatmap (Figure 5D). RT-PCR validation confirmed a significant reduction in *MYC*, *TGFβ2*, *IGFBP3*, and *SERPINE1* in CRE-treated HBECs exposed to VAF347 (Figure 5E). Collectively, these results demonstrate that AhR activation reprograms the allergen-induced transcriptome in HBECs, selectively suppressing senescence-driving pathways and their associated effector genes.

### 3.8. AhR Regulates c-Myc Expression via Direct Promoter Binding

**Figure 6:**
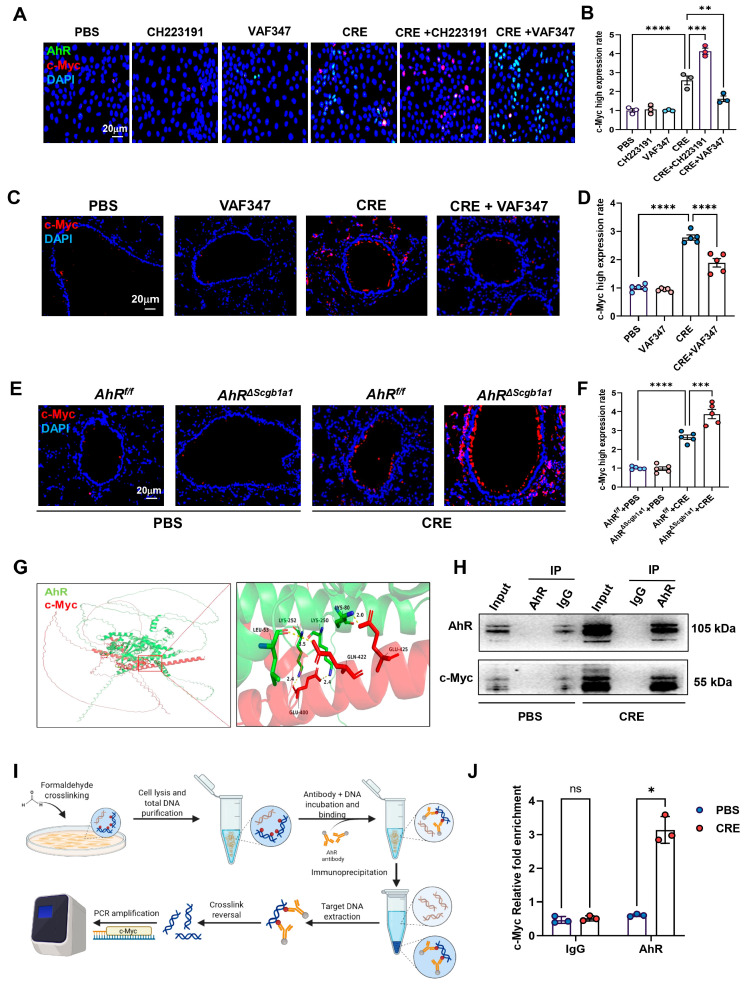
AhR regulates c-Myc expression via direct promoter binding. (**A**) Immunofluorescence staining of c-Myc in HBECs exposed to CRE (20 μg/mL, 24 h) with or without the AhR agonist VAF347 (20 μM) or antagonist CH223191 (10 μM). (**B**) Quantification of c-Myc fluorescence intensity from (**A**). (**C**,**D**) Immunostaining and quantification of MYC expression in lung tissues from CRE-exposed mice treated with or without VAF347. (**E**,**F**) Immunostaining and quantification of MYC expression in lung tissues from CRE-treated *AhR^Scgb1a1^* and *AhR^f/f^* control mice. (**G**) Predicted molecular docking structure showing interaction sites between AhR and c-Myc using the AlphaFold 3 Server platform and visualized using PyMOL (Version 3.0.3). (**H**) Co-immunoprecipitation (Co-IP) of MYC with an anti-AhR antibody. (**I**) Schematic showing the protocol for ChIP-PCR. (**J**) The bindings of AhR to the c-Myc promoter following CRE exposure. *n* = 3. Data represent mean ± SEM; * *p* < 0.05, ** *p* < 0.01, *** *p* < 0.001, **** *p* < 0.0001.

Within the senescence network, the proto-oncogene c-Myc emerged as a central candidate [42,43,44]. To examine whether AhR regulates c-Myc expression, HBECs were exposed to CRE (50 μg/mL, 24 h) in the presence or absence of the AhR agonist VAF347 (20 μM) or antagonist CH223191 (10 μM). c-Myc expression was evaluated by immunostaining (Figure 6A) and quantified (Figure 6B). CRE exposure increased c-Myc expression, which was further elevated by CH223191 and inhibited by VAF347. The VAF347-induced downregulation of c-Myc was confirmed in vivo: lung tissues from CRE-exposed mice treated with VAF347 showed decreased c-Myc expression compared with controls (Figure 6C,D). Conversely, lung tissues from CRE-treated *AhR^ΔScgb1a1^* mice exhibited significantly higher c-Myc expression than those from *AhR^f/f^* controls (Figure 6E,F). To explore a potential regulatory interaction, molecular docking analysis predicted binding interfaces between AhR and c-Myc (Figure 6G). This interaction was experimentally validated by co-immunoprecipitation (Co-IP), where c-Myc was detected in AhR immunoprecipitates (Figure 6H). We further hypothesized that AhR modulates c-Myc transcription via direct promoter binding. Analysis of the JASPAR database identified a conserved AhR-binding motif in the c-Myc promoter. ChIP-PCR using primers flanking this site (Figure 6I) demonstrated significantly enriched AhR binding after CRE exposure (Figure 6J), indicating that AhR directly associates with the c-Myc promoter and regulates its transcriptional activity.

### 3.9. Inhibition of c-Myc Suppresses Allergen-Induced Senescence and SASP Expression in HBECs

**Figure 7:**
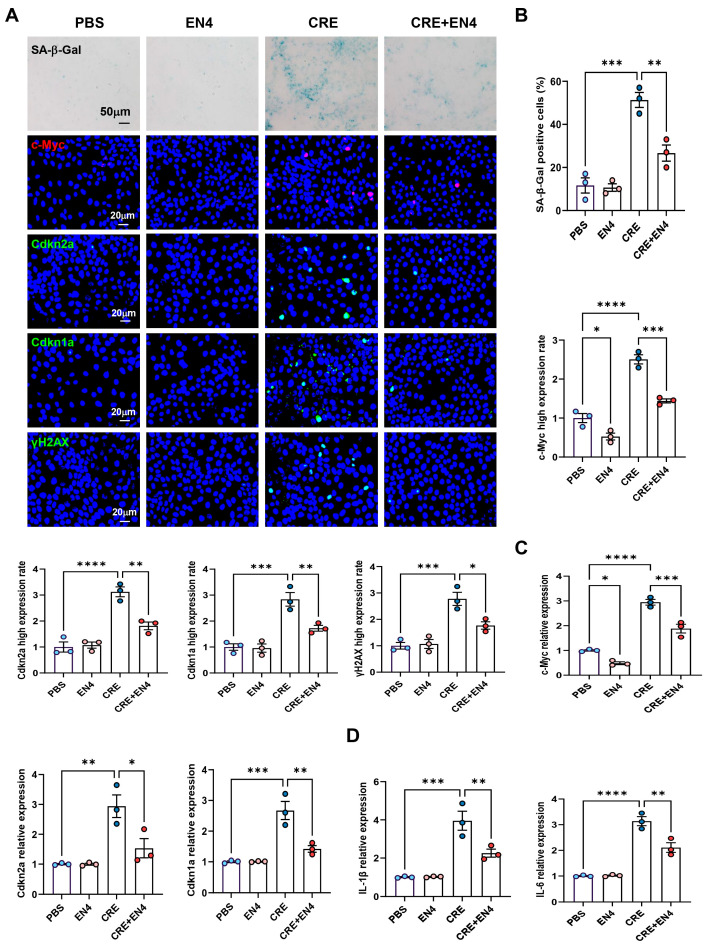
Inhibition of c-Myc suppresses allergen-induced senescence and SASP expression in HBECs. (**A**) Immunofluorescence staining of c-Myc and senescence markers (SA-β-Gal, p16, p21, and γ-H2AX) in HBECs exposed to CRE (20 μg/mL, 24 h) with or without the c-Myc inhibitor EN4 (50 μM). (**B**) Quantification of c-Myc and senescence marker fluorescence intensity from (**A**). (**C**,**D**) RT-PCR analysis of senescence-associated markers (**C**) and SASP-related cytokines (**D**) in HBECs. *n* = 3. Data represent mean ± SEM; * *p* < 0.05, ** *p* < 0.01, *** *p* < 0.001, **** *p* < 0.0001.

Given that c-Myc emerged as a downstream target of AhR, we hypothesized that it contributes to allergen-induced cellular senescence. HBECs were exposed to CRE (50 μg/mL, 24 h) with or without the c-Myc inhibitor EN4 (50 μM). Immunostaining revealed that EN4 effectively suppressed CRE-induced c-Myc expression (Figure 7A,B). Notably, EN4 treatment reduced CRE-induced senescence, as evidenced by decreased SA-β-Gal activity and reduced expression of senescence markers Cdkn2a (p16), Cdkn1a (p21), and γ-H2AX. These inhibitory effects were further confirmed by RT-PCR (Figure 7C). Consistently, EN4 also reduced CRE-induced expression of SASP-related proinflammatory cytokines IL-1β and IL-6 (Figure 7D). These in vitro findings indicate that c-Myc is a functional mediator of allergen-induced cellular senescence and SASP activation.

### 3.10. Inhibiting c-Myc Attenuates Allergen-Induced ROS, Cellular Senescence, and Allergic Airway Inflammation

**Figure 8:**
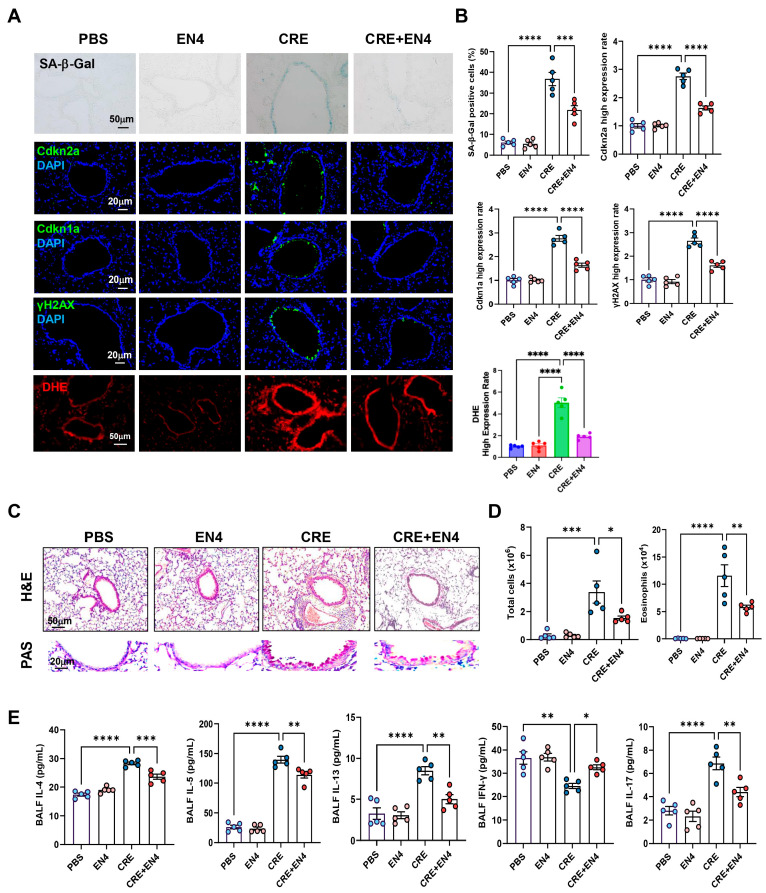
Inhibition of c-Myc attenuates allergen-induced cellular senescence and allergic airway inflammation in vivo. (**A**) Representative immunofluorescence staining for SA-β-Gal activity, senescence-associated markers, and ROS in lung sections. Nuclei were counterstained with DAPI. (**B**) Quantitative analysis of relative fluorescence intensity for SA-β-Gal, Cdkn2a (p16), γ-H2AX, and ROS from images in (**A**). (**C**) Representative images of hematoxylin and eosin staining (H&E, upper) and periodic acid-Schiff staining (PAS, lower) of lung sections. (**D**) Total and eosinophil cell count in bronchoalveolar lavage fluid (BALF). (**E**) BALF cytokine levels measured by ELISA. *n* = 5. Data represent mean ± SEM; * *p* < 0.05, ** *p* < 0.01, *** *p* < 0.001, **** *p* < 0.0001.

Next, we investigated whether c-Myc inhibition mitigates allergen-induced cellular senescence and airway inflammation. To do this, we established a CRE-induced asthma mouse model with or without the c-Myc inhibitor EN4 (Figure S5A). CRE exposure markedly increased SA-β-Gal activity and expression of senescence markers [Cdkn2a (p16), Cdkn1a (p21), and γ-H2AX] in lung tissues, whereas EN4 treatment significantly suppressed these responses (Figure 8A,B). The same pattern was also observed for ROS generation. qRT-PCR analysis confirmed reduced expression of Cdkn2a and Cdkn1a in EN4-treated lungs (Figure S5B). Histological examination revealed that EN4 attenuated peribronchial and perivascular inflammation and decreased mucus hypersecretion (Figure 8C). Consistent with these findings, EN4 lowered total and differential BALF inflammatory cell counts (Figure 8D, Figure S5C), particularly eosinophils, and reduced BALF IL-4, IL-5, IL-13, and IL-17 but increased IFN-γ levels (Figure 8E). Collectively, these results identify c-Myc as a functional mediator of allergen-induced epithelial senescence and airway inflammation.

## 4. Discussion

Here, our study provides new mechanistic insight by showing that cockroach allergen exposure induces excessive ROS production and cellular senescence in bronchial epithelial cells, processes that synergistically amplify airway inflammation. While our study was not to equate chronological aging with senescence, but rather to emphasize that increased epithelial senescence is a key pathological feature commonly associated with aging, and that experimentally inducing or augmenting senescence in young mice provides a relevant model to study its mechanistic contribution to airway inflammation. Importantly, we demonstrate that epithelial AhR plays a critical role in restraining allergen-induced ROS generation, senescence, and inflammation. Furthermore, we identify c-Myc as a novel downstream mediator of AhR signaling, revealing that the AhR-c-Myc axis constitutes a previously unrecognized regulatory pathway controlling epithelial ROS homeostasis, senescence, and airway inflammation, likely in elderly asthmatics.

Cellular senescence has emerged as a fundamental biological process contributing to tissue dysfunction and chronic inflammation in aging-related diseases such as COPD and IPF, largely through its promotion of inflammation and tissue remodeling [8]. Senescence is a complex state marked by cellular stress, DNA damage, irreversible cell cycle arrest, and the secretion of SASP factors [10,11,12]. Given the heterogeneity and context-dependent nature of senescence across cell types and disease stages [8,45,46], a focused investigation is needed to clarify how senescence contributes to allergic airway inflammation and identify potential regulatory pathways and therapeutic targets. To define the cell-type context of senescence in asthma, we analyzed scRNA-seq data from GSE193816 (39,868 endobronchial-brushing cells from allergic asthmatics and allergic controls). The publicly available human scRNA-seq dataset allowed us to evaluate whether signatures of senescence, oxidative stress, and AhR activation observed in our experimental systems were also evident in human airway epithelial cells under allergen challenge. Although limited by sample size, heterogeneity, and lack of longitudinal information, the dataset provided valuable complementary evidence: allergen-exposed asthmatic epithelial cells displayed elevated senescence markers, increased ROS-related gene expression, and enhanced AhR activity. Additionally, our analysis examined epithelial cells as a combined population rather than resolving senescence signatures across individual epithelial subtypes. Because distinct epithelial lineages, such as basal, club, ciliated, and goblet cells, play specialized roles in barrier maintenance, immune crosstalk, and injury responses. Future studies will therefore focus on defining senescence signatures within individual epithelial subtypes and determining how each contributes to allergic airway inflammation.

Given the elevated epithelial ROS and senescence, we hypothesized that these processes amplify inflammatory cascades. Indeed, selective deletion of p16^+^ Club cells markedly attenuated epithelial ROS and senescence, reduced peribronchial and perivascular inflammation, and decreased mucus hypersecretion, BALF total cells, eosinophils, and Th2/17 cytokines, indicating that Club cell-derived ROS and senescence are functional drivers of allergen-induced airway inflammation. We targeted p16^INK4a^-expressing senescent cells because p16 is a well-validated senescence effector and a gold-standard surrogate marker of cellular senescence [47]. Clearance of p16^high^ cells has been shown to ameliorate pathology in multiple organs, including the lung [48]. While our approach targeted p16, it may have been limited in fully identifying and removing all senescent cells. Additionally, Club cells were prioritized because they serve as epithelial progenitors, pollutant and allergen sensors, and key sources of inflammatory mediators [49,50]. Their well-established genetic manipulability (e.g., *Scgb1a1-CreER^T2^*) makes them ideal for dissecting how epithelial ROS and senescence contribute to allergic airway inflammation.

AhR is abundantly expressed in airway tissues and activated by diverse endogenous and exogenous ligands, exerting both pro- and anti-inflammatory effects [51,52,53]. Endogenous ligands such as tryptophan metabolites promote anti-inflammatory responses by inducing IL-10, enhancing CD4^+^FoxP3^+^ Treg generation, and promoting macrophage polarization toward regulatory phenotypes [51]. In allergic models, AhR activation attenuates Th2-driven inflammation and protects against chronic airway diseases such as COPD [54]. Our previous studies demonstrated that epithelial AhR preserves lung homeostasis by controlling autophagy in AT2 cells [28] and suppressing ROS-triggered NLRP3 inflammasome activation that drives mucus hypersecretion [27]. Consistent with these findings, the present study shows that AhR is broadly expressed across lung cell types but is most strongly induced in airway epithelial subsets following allergen challenge, with upregulation confirmed in HBECs and mouse lungs treated with CRE or the AhR agonist VAF347. VAF347, a high-affinity AhR agonist, binds to AhR and initiates canonical AhR-driven transcriptional signaling like the prototype agonist 2,3,7,8-tetrachlorodibenzo-p-dioxin (TCDD) [34,38]. These results underscore the airway epithelium as a central site where AhR integrates environmental cues to restrain ROS generation and modulate downstream immune and inflammatory responses.

Our study extends the protective paradigm of AhR by demonstrating its essential role in suppressing allergen-induced ROS generation and cellular senescence in bronchial epithelial cells and mouse lungs. The coexistence of increased senescence and increased AhR activity in allergic asthma may appear inconsistent with our finding that AhR activation protects against allergen-induced senescence. It is likely that the elevated AhR activity we observed in asthma likely reflects a compensatory response to allergen-induced epithelial stress, but this activation is often incomplete, dysregulated, or unable to counteract the strong pro-senescent signals, resulting in the coexistence of high senescence and modest endogenous AhR activation. This interpretation is supported by the fact that AhR-deficient mice exhibit elevated ROS accumulation, enhanced epithelial senescence, worsened inflammation, indicating that AhR is essential for maintaining airway homeostasis. Moreover, pharmacologic AhR agonists enhance this protective pathway beyond endogenous levels, thereby suppressing inflammation. These findings provide direct evidence that epithelial AhR functions as a key regulator restraining allergen-induced oxidative stress and senescence, thereby maintaining epithelial integrity and limiting allergic airway inflammation. However, environmental influences on the airway epithelium are mediated not only through pathways such as AhR signaling and oxidative stress, but also through epigenetic mechanisms including DNA methylation, histone modifications, and chromatin remodeling [55,56,57,58]. These processes collectively shape epithelial vulnerability to injury, modulate inflammatory responses, and influence long-term disease trajectories. Notably, epigenetic modifications within the airway epithelium are altered in asthma, respond dynamically to inflammatory cytokines, and correlate with disease-relevant transcriptional programs. They also impact pathways central to asthma pathology, including epithelial barrier integrity, airway remodeling/fibrotic responses, epithelial–mesenchymal transition (EMT), and downstream immune signaling [55,56,59,60]. Future studies are needed to determine how AhR-mediated signaling intersects with these broader epigenetic programs to fully define the spectrum of environmental effects on epithelial biology in asthma. Mechanistically, transcriptomic analysis revealed that AhR activation profoundly reprograms the allergen-induced epithelial response. Pharmacologic stimulation with VAF347 strongly induced canonical AhR targets (*AHRR*, *CYP1B1*, *CYP1A1*), confirming effective pathway engagement, while broadly suppressing allergen-driven transcriptional programs. Pathway enrichment analysis identified cellular senescence among the most significantly altered pathways, along with focal adhesion, tight junction, and extracellular matrix–receptor interactions. Within the senescence network, the proto-oncogene c-Myc emerged as a central regulatory node, consistent with previous evidence linking AhR to c-Myc regulation [42,43,44]. We confirmed this relationship through complementary in vitro and in vivo approaches. In HBECs, CRE-induced c-Myc expression and ROS generation were both reduced by VAF347 and enhanced by the AhR antagonist CH223191, indicating that AhR negatively regulates c-Myc and its associated oxidative stress program. Molecular docking and co-immunoprecipitation confirmed a physical association between AhR and c-Myc, and ChIP-PCR demonstrated AhR occupancy at a conserved motif within the c-Myc promoter after allergen exposure.

c-Myc, a well-characterized proto-oncogene and transcription factor, orchestrates diverse cellular processes including proliferation, metabolism, and apoptosis [61]. Beyond these canonical roles, c-Myc maintains stemness by promoting cancer stem cell self-renewal, thereby contributing to tumorigenesis and chronic inflammation [62,63,64]. Importantly, c-Myc also modulates cellular senescence—a tumor-suppressive program that limits uncontrolled proliferation. Environmental carcinogens such as benzo [a]pyrene (BaP) can stabilize c-Myc and suppress senescence, driving oncogenic programs associated with growth, metastasis, and therapy resistance [43]. Notably, c-Myc is overexpressed in peripheral group 2 innate lymphoid cells (ILC2s) from asthma patients, where it enhances type 2 inflammation, and its inhibition dampens allergen-induced type 2 responses [65]. Consistent with these findings, our study shows that c-Myc is upregulated in CRE-treated HBECs and mouse lungs, linking allergen exposure to epithelial ROS generation and senescence. Particularly, pharmacologic inhibition of c-Myc with EN4 effectively suppressed epithelial senescence, SASP secretion (e.g., *IL-1β*, *IL-6*), ROS production, and airway inflammation. These results identify c-Myc as a functional mediator linking AhR activation to epithelial ROS regulation, senescence, and downstream inflammatory responses.

This study demonstrates that allergen exposure induces epithelial ROS generation and senescence, establishing senescence as an active driver of airway inflammation through SASP-mediated cytokine release. We identify the AhR-c-Myc signaling axis as a key regulator of this process, providing a direct mechanistic link between environmental sensing and pathological epithelial responses. Pharmacologic inhibition of c-Myc effectively reduced ROS accumulation, epithelial senescence, airway inflammation, and Th2/Th17 cytokine production, underscoring the translational potential of targeting this pathway. Nonetheless, certain limitations should be acknowledged. First, senescence dynamics under chronic or repeated allergen exposure remain incompletely defined and may differ from the acute responses characterized here. Second, while our data implicates c-Myc as a downstream effector of AhR, epithelial-specific genetic approaches will be required to confirm its causal role. Third, deeper mechanistic studies are needed to delineate how AhR-c-Myc signaling orchestrates epithelial senescence and modulates immune responses. Additionally, our analysis did not resolve epithelial heterogeneity, and distinct epithelial subtypes may differentially contribute to senescence-driven inflammation. Finally, our mouse models primarily reflect T2-high allergic asthma, and thus, the epithelial ROS-senescence-AhR-c-Myc axis described here may not fully translate to non-T2 or steroid-resistant forms of the disease.

## Data Availability

The original data presented in the study are openly available in the Gene Expression Omnibus (GEO) database at accession number GSE313889.

## Supplementary Materials

The following supporting information can be downloaded at https://www.mdpi.com/article/10.3390/antiox15010022/s1, Figure S1. Genetic identification of *p16^ΔScgb1a1^* mice. (A) Schematic diagram depicts the generation of *p16^ΔScgb1a1^* mice by crossing *p16^f/f^* mice with *Scgb1a1-CreER^TM^* mice. (B) Specific depletion of p16^+^ cells in Club cells was confirmed by genotyping of mouse lung tissues. (C) Differential immune cells in bronchoalveolar lavage fluid (BALF). *n* = 5. Data represent mean ± SEM; * *p* < 0.05, ** *p* < 0.01, **** *p* < 0.0001. Figure S2. AhR signaling regulates allergen-induced senescence in HBECs. (A) Immunofluorescence staining of senescence markers. (B) Quantification of c-Myc and senescence marker fluorescence intensity from (A). (C,D) RT-PCR analysis of senescence-associated markers (C) and SASP-related cytokines (D) in HBECs. *n* = 3. Data represent mean ± SEM; * *p* < 0.05, ** *p* < 0.01, *** *p* < 0.001, **** *p* < 0.0001. Figure S3. Genetic identification of AhRΔScgb1a1 mice. (A) Schematic diagram depicts the generation of AhRΔScgb1a1 mice by crossing AhRf/f mice with Scgb1a1-CreERTM mice. (B,D) Specific depletion of AhR^+^ cells in Club cells was confirmed by genotyping (B) and immunostaining (C) of mouse lung tissues. (D) RT-PCR analysis of AhR and senescence-associated gene expression. (E) Differential immune cells in bronchoalveolar lavage fluid (BALF). *n* = 5. Data represent mean ± SEM; * *p* < 0.05, ** *p* < 0.01, *** *p* < 0.001, **** *p* < 0.0001. Figure S4. AhR agonist VAF347 suppresses cockroach allergen-induced senescence and airway inflammation. (A) Experimental protocol for the cockroach extract (CRE)-induced asthma mouse model with or without treatment with the AhR agonist VAF347. (B) Representative immunofluorescence staining for SA-β-Gal activity and senescence-associated markers in lung sections. Nuclei were counterstained with DAPI. (C) Quantitative analysis of relative fluorescence intensity from images in (B). (D) Quantitative RT-PCR analysis of senescence-associated markers in lung tissues. (E) Representative images of hematoxylin and eosin staining (H&E, upper) and periodic acid–Schiff staining (PAS, lower) of lung sections. (F) Total and eosinophil cell count in bronchoalveolar lavage fluid (BALF). (G) BALF cytokine levels measured by ELISA. *n* = 5. Data represent mean ± SEM; * *p* < 0.05, ** *p* < 0.01, *** *p* < 0.001, **** *p* < 0.0001. Figure S5. Inhibition of c-Myc attenuates allergen-induced cellular senescence and allergic airway inflammation in vivo. (A) Experimental scheme of mouse asthma model. (B) RT-PCR analysis of senescence-associated gene expression. (C) Differential immune cells in bronchoalveolar lavage fluid (BALF). *n* = 5. Data represent mean ± SEM; * *p* < 0.05, ** *p* < 0.01, **** *p* < 0.0001. Table S1. Demographic and clinical characteristics of participating subjects. Table S2. Flow cytometric antibody. Table S3. Antibodies used for western blot and immunofluorescence staining. Table S4. Primers for qRT-PCR.

## Abbreviations

CRE	Cockroach extract
BALF	Bronchoalveolar lavage fluid
AhR	Aryl hydrocarbon receptor
SASP	Senescence-associated secretory phenotype
ROS	Reactive oxygen species
RNA-seq	RNA sequencing
scRNA-seq	Single-cell RNA-seq
HBEC	Human bronchial epithelial cell
DEG	Differentially expressed genes
GSEA	Gene set enrichment analysis
KEGG	Kyoto encyclopedia of genes and genomes
Cdkn2a	Cyclin dependent kinase inhibitor 2A
Cdkn1a	Cyclin dependent kinase inhibitor 1A
IP	Immunoprecipitation
ChIP	Chromatin immunoprecipitation
qRT-PCR	Quantitative reverse transcription-polymerase chain reaction
SA-β-Gal	Senescence β-Galactosidase
OCT	Optimal cutting temperature
DAPI	4′,6-diamidino-2-phenylindole
ANOVA	An ordinary one-way analysis of variance
