# Supplementary material for: Epithelial AhR Suppresses Allergen-Induced Oxidative Stress and Senescence via c-Myc Regulation
Source: Antioxidants (Basel). 2025 Dec 23;15(1):22. doi: 10.3390/antiox15010022 (PMC12837779; doi:10.3390/antiox15010022)
Supplement: Supplementary file 1 [file antioxidants-15-00022-s001.zip › antioxidants-3970518-supplementary.pdf]

METHODS

Human model and bronchoscopic segmental allergen challenge (SAC)

Raw count matrices for human asthma models were retrieved from the Gene Expression Omnibus (accession GSE1938161). All samples were obtained in the original study under protocols approved by the institutional review boards of Massachusetts General Hospital, and that all participants provided written informed consent. Detailed demographic and clinical characteristics of the participating subjects were included in Supplementary Table S1. Adult participants with allergy to house dust mite or cat underwent a standardized SAC protocol in the previous publication. After baseline bronchoscopy, bronchoalveolar lavage (BAL) and endobronchial brushings were obtained from a mid-lung segment. During the same procedure, one airway segment was instilled with diluent (saline), and another segment with allergen. A repeat bronchoscopy was performed 24 hours later to collect BAL and endobronchial brush samples from the diluent- and allergen-challenged segments. This model recapitulates features of an allergen-induced asthma exacerbation while limiting exposure to a localized airway region.

Endobronchial brushing and initial cell handling

Endobronchial brushings from third–fourth generation airways were collected into ice-cold phenol-red-free RPMI-1640 supplemented with 2% human AB serum and a ROCK inhibitor and processed within approximately 60 minutes of collection. Cells were gently dislodged from the cytology brushes, resuspended, and passed through filters to obtain single-cell suspensions.

Viability and enrichment steps

To maximize cell quality for scRNA-seq, dead cells were depleted using annexin V-based magnetic beads and residual red blood cells were reduced with an antibody against glycophorin A. Cell viability was assessed by trypan blue staining before and after these steps; samples used for library preparation had post-enrichment viabilities >95%. Live cells were finally resuspended at ~800–

1000 viable cells/μL in preparation for single-cell capture.

Single-cell capture, library preparation and sequencing

Single-cell 3′ expression libraries were generated using a droplet-based microfluidic platform (Chromium Single Cell 3′ v3 chemistry, 10x Genomics) according to the manufacturer's protocol for endobronchial brush samples. Libraries were sequenced on an Illumina HiSeq X Ten instrument to a depth sufficient for robust detection of airway epithelial and immune cell populations.

Primary processing and generation of expression matrices

The original authors performed read alignment to the human reference genome (GRCh38), UMI counting, and initial quality control using standard single-cell RNA-seq pipelines, resulting in the gene–cell count matrices that are publicly available at GEO (GSE193816). In our study, we did not repeat any wet-lab procedures; all analyses began from these processed expression matrices and their associated cell-level metadata (e.g. subject ID, group, condition, and broad cell type annotations).

Dimensionality reduction and visualization

Uniform Manifold Approximation and Projection (UMAP) embedded the data in two dimensions for visualization; the first two UMAP components are shown in relative figures. Pre-Annotated information by original study was used for cell type identification.

Quantification of cellular senescence

To score the senescence programme in individual cells, we employed AUCell from irGSEA package with the SenMayo senescence signature which was the best gene set for estimate the senescence levels with high consistency to experimental results. For each cell, AUCell computes the area under the cumulative recovery curve of the ranked gene-expression list; the resulting

enrichment score was used as a quantitative proxy for senescence burden.

Inference of AHR transcriptional activity

Regulons for the aryl hydrocarbon receptor (AHR) were extracted from the DoRothEA v2 knowledgebase, limited to confidence levels A–C to ensure high-quality interactions. Transcriptomic activity was inferred with the VIPER algorithm using the scale mode, which normalises enrichment scores across cells and allows direct comparison between conditions[8].

Statistical testing

Differentially expressed genes (DEGs) between AA_Ag and AC_Ag cells within each major cell type were identified with the Wilcoxon rank-sum test. The same non-parametric test compared (i) AUCell senescence scores and (ii) VIPER-derived AhR activity scores across clinical groups. P-values < 0.05 was considered significant.

Senescence-associated β-galactosidase staining

The cells and sections were fixed with the fixative solution from the kit at room temperature for half an hour. After washing with PBS, the SA-β-gal staining solution was added, and the samples were incubated overnight at 37 °C in a CO_2-free environment. After incubation, the staining working solution was aspirated, and were gently washed with PBS. They were observed under a NIKON ECLIPSE Ti-U microscope (Nikon, USA) equipped with a DS-Fi2 camera.

Co-immunoprecipitation analysis and western Blotting

After the intervention, the HBECs were lysed with radioimmunoprecipitation assay (RIPA) buffer containing a mixture of protease and phosphatase inhibitors (Sigma-Aldrich), and the protein supernatant was obtained by centrifugation. The protein concentration was measured using a BCA kit (Thermo Fisher). The proteins were incubated with 5 μg of antibody or IgG-coupled protein overnight at 4°C. The next day, the incubated immunoprotein complexes were incubated with

Protein A/G Magnetic Beads (HY-K0202, MCE) for 2 hours at 4°C. Then, the magnetic beads were washed six times with immunoprecipitation lysis buffer (HY-K1000, MCE). After washing, the magnetic beads were resuspended in LDS Sample Buffer (Thermo Fisher) to elute the bound proteins. The eluted proteins were loaded onto a 4%-12% Tris-glycine gel in NuPAGE MOPS SDS electrophoresis buffer (Thermo Fisher) for electrophoresis. Transfer was carried out using the iBlot2 NC stack system (Thermo Fisher). The membrane was blocked in 5% BSA in TBST for 1 hour at room temperature, then incubated with the primary antibody overnight at 4°C and coupled with an appropriate secondary antibody. Subsequently, chemiluminescent gel imaging was performed using the iBright 1500 imaging system (Thermo Fisher) to capture the images.

Table S1. Demographic and clinical characteristics of participating subjects

Variable	AC (n = 4)	AA (n = 4)	P value
Age, years	27.5 (21.5-36.5)	25.0 (21.8-32.0)	0.886
Female, No. (%)	4 (100.0)	3 (75.0)	0.285
Ethnicity, No. (%)			0.465
Hispanic or Latino	2 (50.0)	1 (25.0)	
Not Hispanic or Latino	2 (50.0)	3 (75.0)	
Allergen, No. (%)			0.028
Cat	3 (75.0)	0 (0.0)	
DP	1 (25.0)	4 (100.0)	
Allergen, (BAU or AU)	246.7 (61.6-925.8)	13.7 (6.9-13.7)	0.029
FEV1 (L)	3.2 (2.7-3.5)	3.3 (3.0-3.9)	0.486
FEV1 (% predicted)	100.5 (97.0-105.5)	95.0 (90.8-110.5)	0.486
FVC (L)	3.5 (3.0-3.9)	3.7 (3.6-5.2)	0.686
FVC (% predicted)	96.0 (89.3-105.0)	101.0 (98.5-105.0)	0.686

Table S2. Flow cytometric antibody

Antibody	Cat.	Company	Country
TruStain FcX™ PLUS (anti-mouse CD16/32) Antibody	156604	Biolegend	USA
PE anti-mouse CD170 (Siglec-F) Antibody	155506	Biolegend	USA
PE Mouse IgG2a, κ Isotype Ctrl Antibody	400212	Biolegend	USA
FITC anti-mouse CD107b (Mac-3) Antibody	108504	Biolegend	USA
FITC Mouse IgG2a, κ Isotype Ctrl Antibody	400208	Biolegend	USA
APC anti-mouse Ly-6G/Ly-6C (Gr-1) Antibody	108412	Biolegend	USA
PC Mouse IgG2a, κ Isotype Ctrl Antibody	400220	Biolegend	USA
PerCP/Cyanine5.5 anti-mouse CD3ε Antibody	100328	Biolegend	USA
PerCP/Cyanine5.5 Mouse IgG2a, κ Isotype Ctrl Antibody	400258	Biolegend	USA

Table S3. Antibodies used for western blot and immunofluorescence staining

Target	Species	Clone	Assay (dilution)	Company
AHR	Rabbit	GTX22770	IF (1:100)	GeneTex
			WB (1:1000)	
MYC	Mouse	60003-2-Ig	IF (1:100)	Proteintech
			WB (1:1000)	
CDKN2A/P16-INK4A	Rabbit	10883-1-AP	IF (1:100)	Proteintech
p21	Rabbit	2H2L13	IF (1:100)	Invitrogen
H2A.X	Rabbit	7631S	IF (1:100)	Cell Signaling Technology
DAPI	-	62248	IF (1:5000)	Invitrogen

Table S4. Primers for qRT-PCR

Gene	Species	NCBI GeneID	Sequence (5'-3')
AhR	Mus musculus	11622	Fwd: GCCGGTGCAGAAAACAGTAAA<br>Rev: GGTAACTGACGCTGAGCCTA
c-Myc	Mus musculus	17869	Fwd: CACCAGCAGCGACTCTGAAGAAG<br>Rev: GCCCGACTCCGACCTCTTGG
p16	Mus musculus	12578	Fwd: TTCCGCTGGGTGCTCTTTGTG<br>Rev: GAAGCTATGCCCGTCGGTCTG
P21	Mus musculus	12575	Fwd: CCTTGTCGCTGTCTTGCACTCTG<br>Rev: GCTGGTCTGCCTCCGTTTTCG
IL-1β	Mus musculus	16176	Fwd: AATCTCGCAGCAGCACATCAAC<br>Rev: AGGTCCACGGGAAAGACACAG
IL-6	Mus musculus	16193	Fwd: GAGAGGAGACTTCACAGAGGATACC<br>Rev: TCATTTCCACGATTTCCCAGAGAAC
β-actin	Mus musculus	11461	Fwd: GTGCTATGTTGCTCTAGACTTCG<br>Rev: ATGCCACAGGATTCCATACC
AhR	Homo sapiens	196	Fwd: ACATCACCTACGCCAGTCGC<br>Rev: TCTATGCCGCTTGGAAGGAT
c-Myc	Homo sapiens	4609	Fwd: CGTCCTCGGATTCTCTGCTCTC<br>Rev: GGTGGTGGGCGGTGTCTC
IGFBP3	Homo sapiens	3486	Fwd: AAGCAGTGTCGCCCTTCCAAAG<br>Rev: TTGGTGGTGTAGCCTGGGAGAG
TGF-β2	Homo sapiens	7042	Fwd: ACAAGAGCAGAAGGCGAATGGC<br>Rev: GTGCAGCAGGGACAGTGTAAGC
SERPINE1	Homo sapiens	5054	Fwd: CCGCCGCCTCTTCCACAAATC<br>Rev: AGGGCAGTTCCAGGATGTCGTAG
p16	Homo sapiens	1029	Fwd: GCCCAACGCACCGAATAGTTAC<br>Rev: ACGGGTCGGGTGAGAGTGG
P21	Homo sapiens	1026	Fwd: ACCCTTGTGCCTCGCTCAG<br>Rev: GGTAGAAATCTGTCATGCTGGTCTG
IL-1β	Homo sapiens	3553	Fwd: GGACAGGATATGGAGCAACAAGTGG<br>Rev: TCATCTTTCAACACGCAGGACAGG
IL-6	Homo sapiens	3569	Fwd: GCCTTCGGTCCAGTTGCCTTC<br>Rev: GTTCTGAAGAGGTGAGTGGCTGTC
GAPDH	Homo sapiens	2597	Fwd: CAGGAGGCATTGCTGATGAT<br>Rev: GAAGGCTGGGGCTCATTT
c-Myc	Homo sapiens	4609	Fwd: TTGTGTAGTGACCATTCTAGGCA<br>Rev: GGATTTAAGGAAGGCAGCGGA

FIGURE LEGENDS

Figure S1. Genetic identification of *p16*$^{\Delta Scgb1a1}$ mice. (**A**) Schematic diagram depicts the generation of *p16*$^{\Delta Scgb1a1}$ mice by crossing *p16*$^{f/f}$ mice with *Scgb1a1-CreER*^{TM} mice. (**B**) Specific depletion of p16^{+} cells in Club cells was confirmed by genotyping of mouse lung tissues. (**C**) Differential immune cells in bronchoalveolar lavage fluid (BALF). n=5. Data represent mean ± SEM; *$p < 0.05$, **$p < 0.01$, ****$p < 0.0001$.*

Figure S2. AhR signaling regulates allergen-induced senescence in HBECs. (**A**) Immunofluorescence staining of senescence markers. (**B**) Quantification of c-Myc and senescence marker fluorescence intensity from (**A**). (**C-D**) RT-PCR analysis of senescence-associated markers (**C**) and SASP-related cytokines (**D**) in HBECs. n=3. Data represent mean ± SEM; *$p < 0.05$, **$p < 0.01$, ***$p < 0.001$, ****$p < 0.0001$.*

Figure S3. Genetic identification of AhRΔScgb1a1 mice. (A) Schematic diagram depicts the generation of AhRΔScgb1a1 mice by crossing AhRf/f mice with Scgb1a1-CreERTM mice. (B-D) Specific depletion of AhR^{+} cells in Club cells was confirmed by genotyping (B) and immunostaining (C) of mouse lung tissues. (D) RT-PCR analysis of AhR and senescence-associated gene expression. (E) Differential immune cells in bronchoalveolar lavage fluid (BALF). n=5. Data represent mean ± SEM; *$p < 0.05$, **$p < 0.01$, ***$p < 0.001$, ****$p < 0.0001$.*

Figure S4. AhR agonist VAF347 suppresses cockroach allergen–induced senescence and airway inflammation. (**A**) Experimental protocol for the cockroach extract (CRE)–induced asthma mouse model with or without treatment with the AhR agonist VAF347. (**B**) Representative immunofluorescence staining for SA-β-Gal activity and senescence-associated markers in lung sections. Nuclei were counterstained with DAPI. (**C**) Quantitative analysis of relative fluorescence intensity from images in (B). (**D**) Quantitative RT-PCR analysis of senescence-associated markers in lung tissues. (**E**) Representative images of hematoxylin and eosin staining (H&E, upper) and periodic acid–Schiff staining (PAS, lower) of lung sections. (**F**) Total and eosinophil cell count in bronchoalveolar lavage fluid (BALF). (**G**) BALF cytokine levels measured by ELISA. n=5. Data represent mean ± SEM; **p < 0.05, **p < 0.01, ***p < 0.001, ****p < 0.0001.*

Figure S5. Inhibition of c-Myc attenuates allergen-induced cellular senescence and allergic airway inflammation in vivo. (**A**) Experimental scheme of mouse asthma model. (**B**) RT-PCR analysis of senescence-associated gene expression. (**C**) Differential immune cells in bronchoalveolar lavage fluid (BALF). n=5. Data represent mean ± SEM; **p < 0.05, **p < 0.01, ****p < 0.0001.*

Figure S1

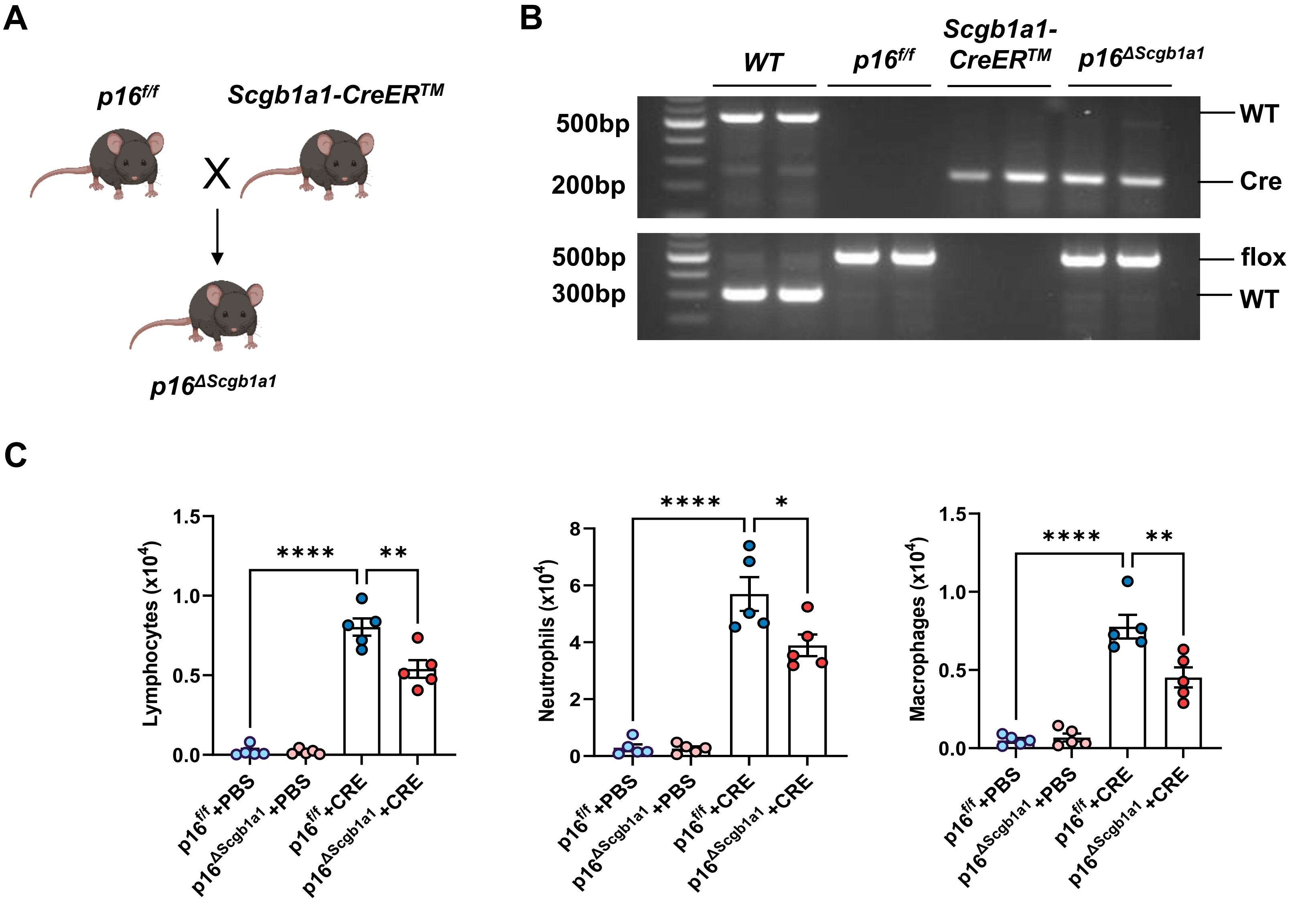

A
p16f/f
Scgb1a1-CreERTM
X
p16ΔScgb1a1
B
WT
p16f/f
Scgb1a1-
CreERTM
p16ΔScgb1a1
500bp
200bp
WT
Cre
500bp
300bp
flox
WT
C
Lymphocytes (x10⁴)
1.5
1.0
0.5
0.0
****
**
Neutrophils (x10⁴)
8
6
4
2
0
****
*
Macrophages (x10⁴)
1.5
1.0
0.5
0.0
****
**
p16f/f +PBS
p16ΔScgb1a1 +PBS
p16f/f +CRE
p16ΔScgb1a1 +CRE

Figure S2

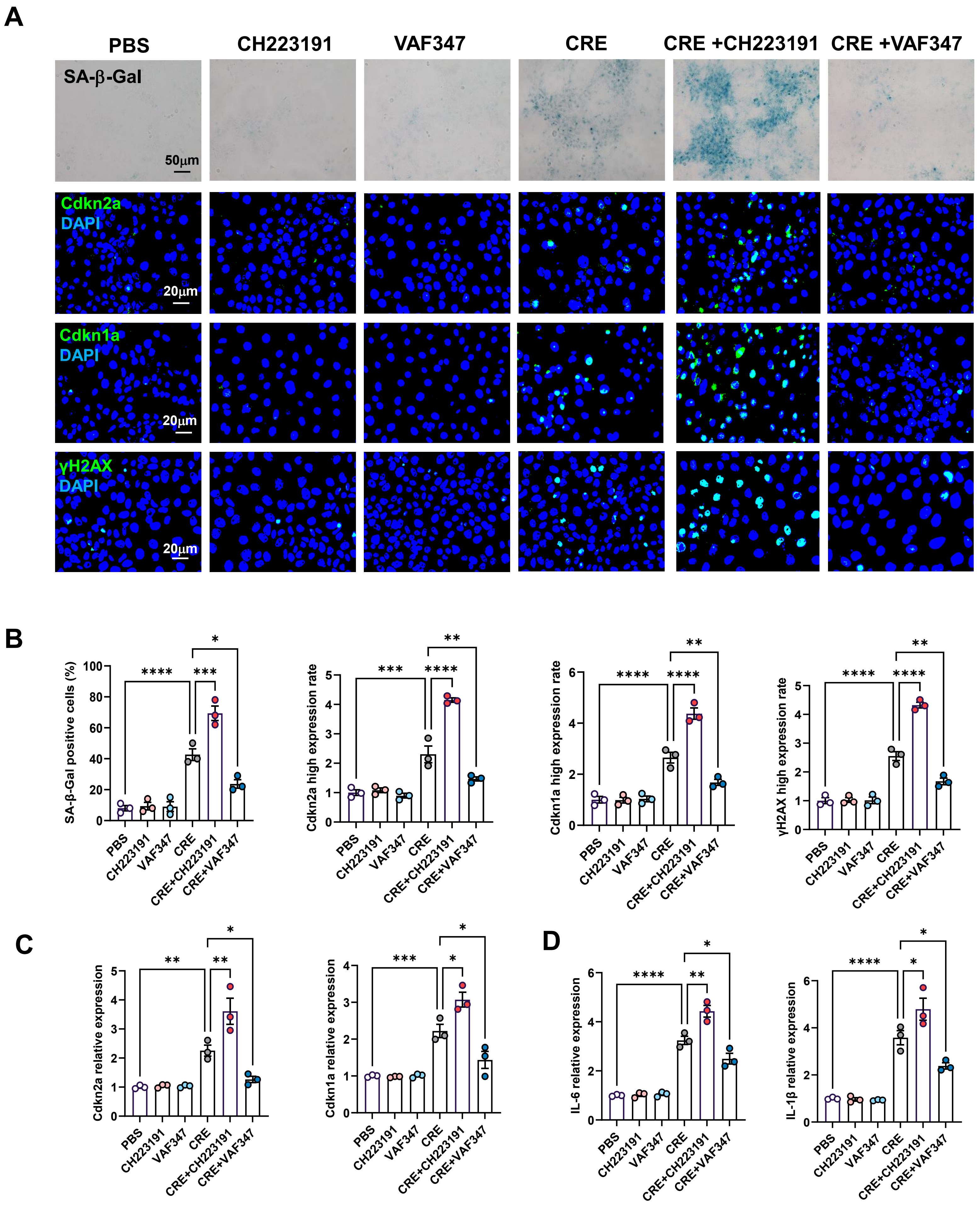
A
PBS
CH223191
VAF347
CRE
CRE +CH223191
CRE +VAF347
SA-β-Gal
50μm
Cdkn2a
DAPI
20μm
Cdkn1a
DAPI
20μm
γH2AX
DAPI
20μm
B
SA-β-Gal positive cells (%)
Cdkn2a high expression rate
Cdkn1a high expression rate
γH2AX high expression rate
PBS
CH223191
VAF347
CRE
CRE+CH223191
CRE+VAF347
C
Cdkn2a relative expression
Cdkn1a relative expression
D
IL-6 relative expression
IL-1β relative expression

Figure S3

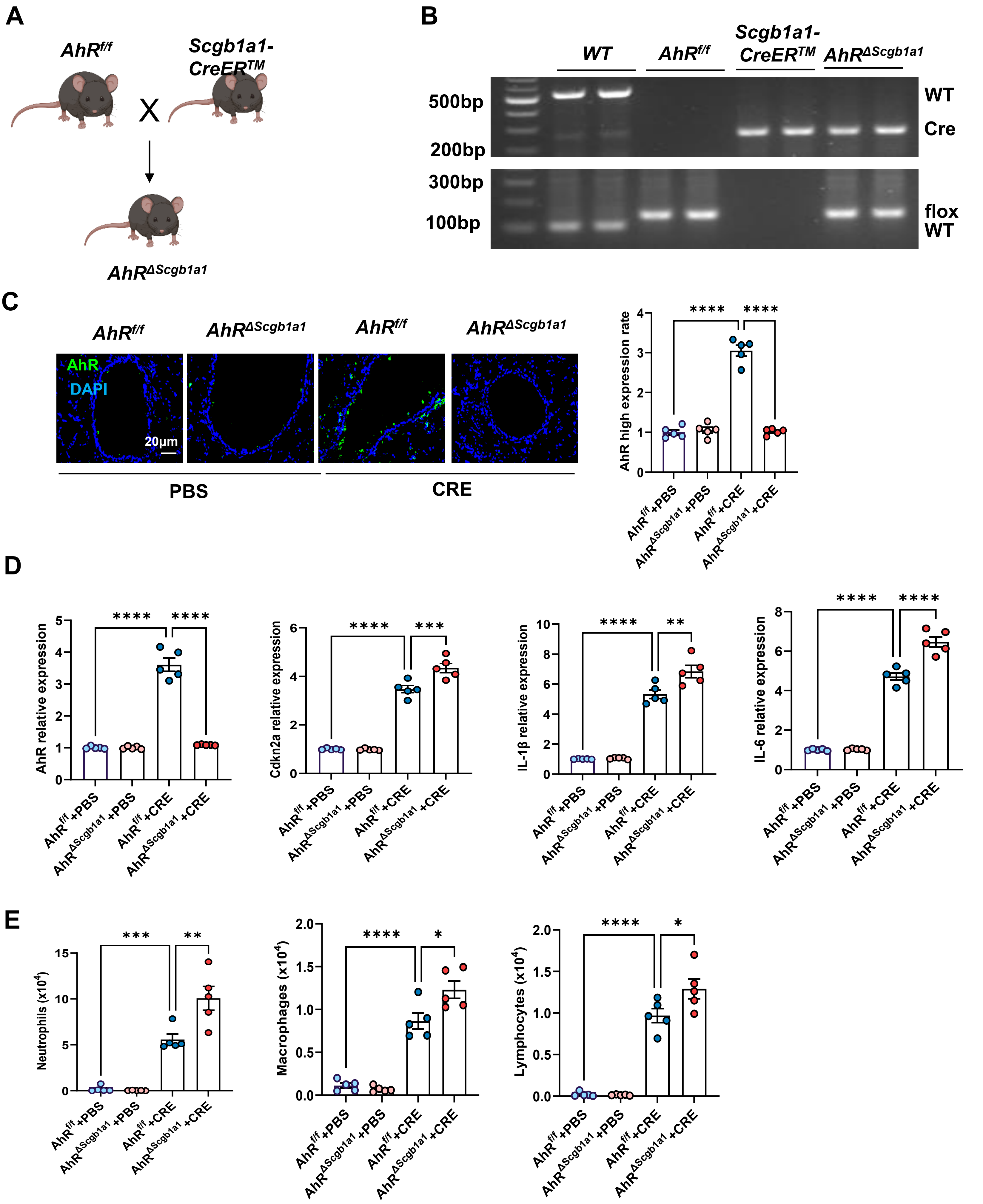
A
AhRf/f
Scgb1a1-CreERTM
X
AhRΔScgb1a1
B
WT
AhRf/f
Scgb1a1-CreERTM
AhRΔScgb1a1
500bp
200bp
300bp
100bp
WT
Cre
flox
WT
C
AhRf/f
AhRΔScgb1a1
AhRf/f
AhRΔScgb1a1
AhR
DAPI
20μm
PBS
CRE
AhR high expression rate
AhRf/f +PBS
AhRΔScgb1a1 +PBS
AhRf/f +CRE
AhRΔScgb1a1 +CRE
D
AhR relative expression
Cdkn2a relative expression
IL-1β relative expression
IL-6 relative expression
E
Neutrophils (x10^4)
Macrophages (x10^4)
Lymphocytes (x10^4)

Figure S4

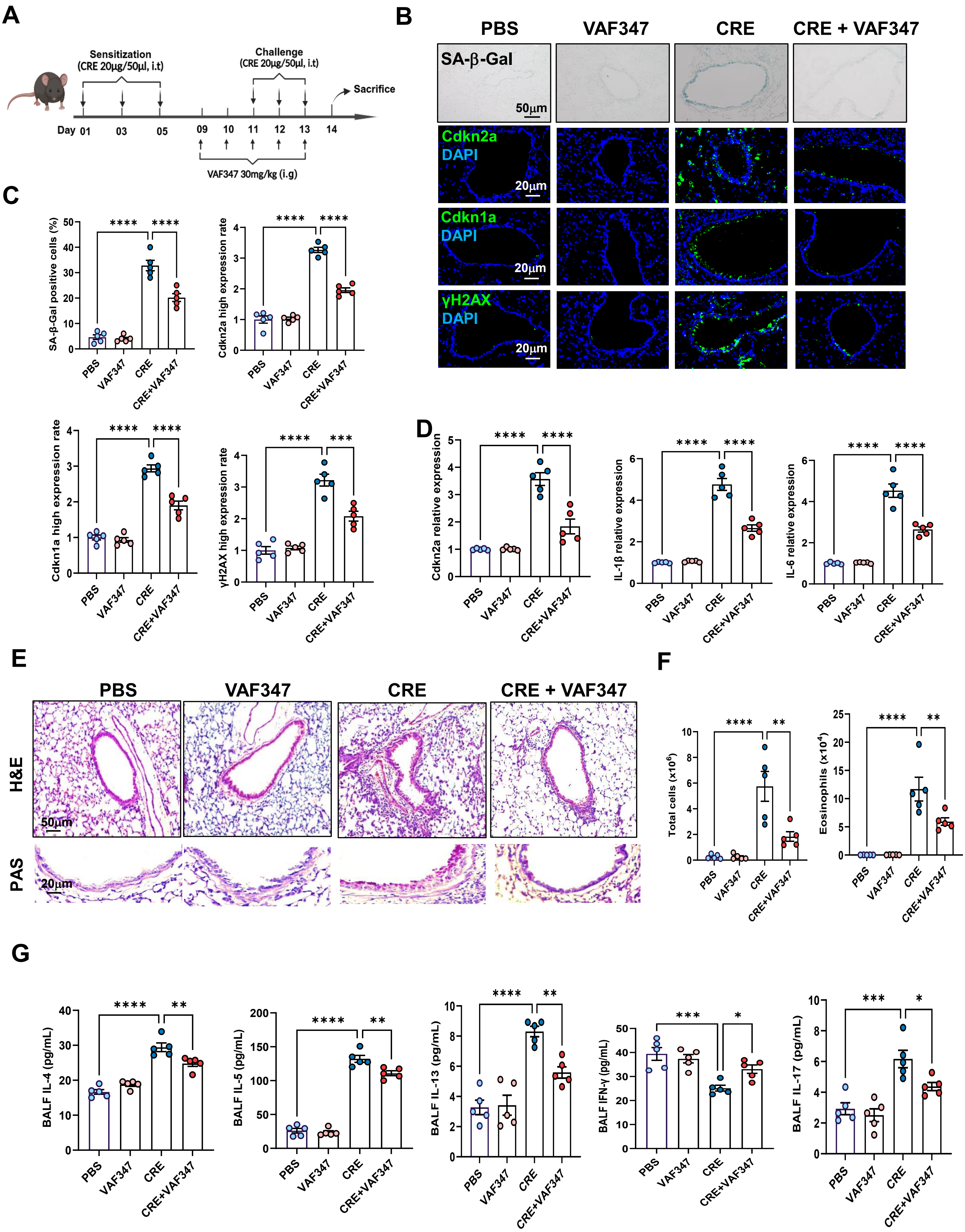

A
Sensitization (CRE 20μg/50μl, i.t)
Challenge (CRE 20μg/50μl, i.t)
Sacrifice
Day 01 03 05 09 10 11 12 13 14
VAF347 30mg/kg (i.g)
B
PBS
VAF347
CRE
CRE + VAF347
SA-β-Gal
50μm
Cdkn2a DAPI
20μm
Cdkn1a DAPI
20μm
γH2AX DAPI
20μm
C
SA-β-Gal positive cells (%)
Cdkn2a high expression rate
Cdkn1a high expression rate
γH2AX high expression rate
D
Cdkn2a relative expression
IL-1β relative expression
IL-6 relative expression
E
PBS
VAF347
CRE
CRE + VAF347
H&E
50μm
PAS
20μm
F
Total cells (x10^6)
Eosinophils (x10^4)
G
BALF IL-4 (pg/mL)
BALF IL-5 (pg/mL)
BALF IL-13 (pg/mL)
BALF IFN-γ (pg/mL)
BALF IL-17 (pg/mL)
PBS
VAF347
CRE
CRE+VAF347

Figure S5

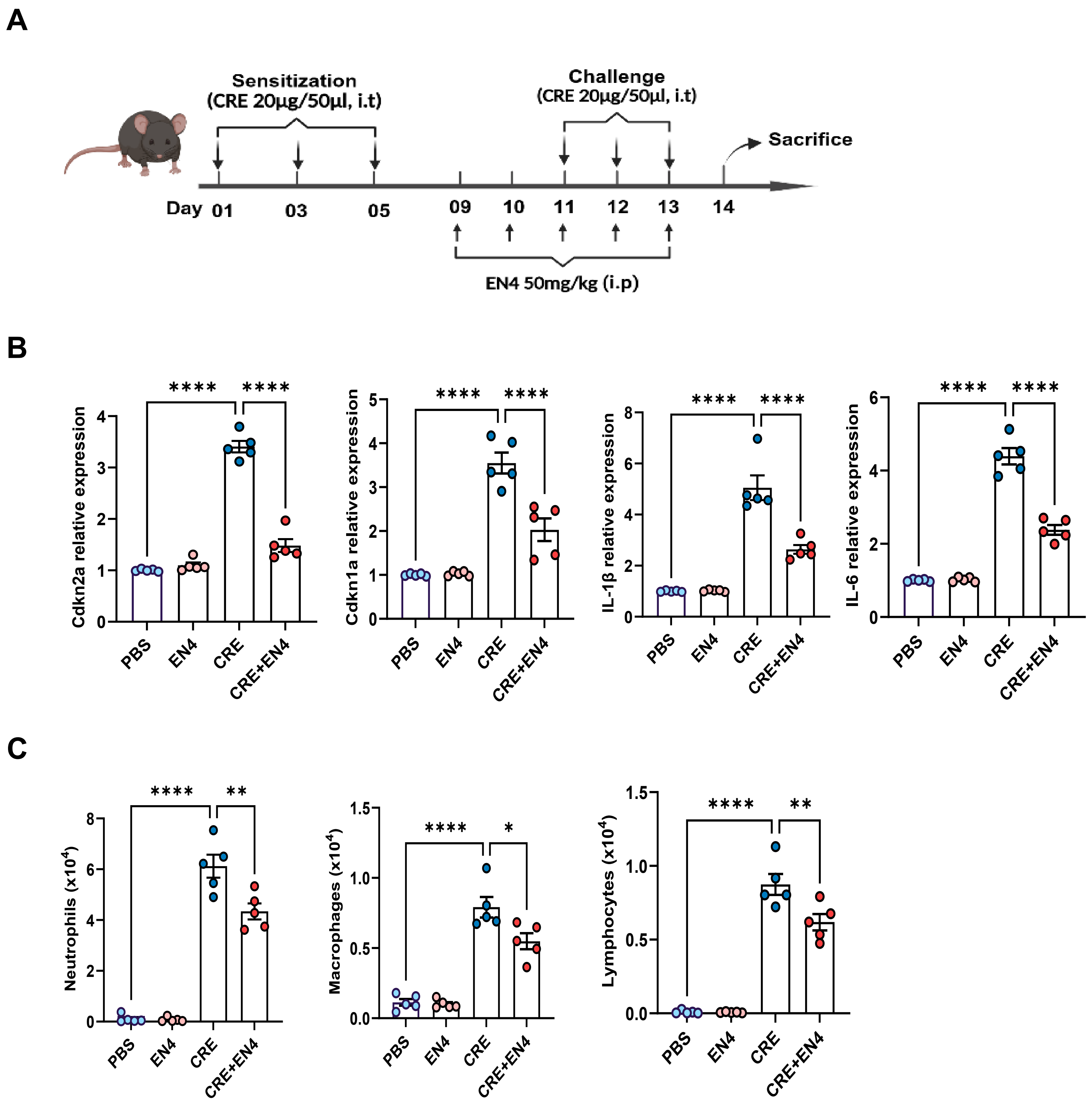
A
Sensitization
(CRE 20µg/50µl, i.t)
Challenge
(CRE 20µg/50µl, i.t)
Sacrifice
Day 01 03 05 09 10 11 12 13 14
EN4 50mg/kg (i.p)
B
Cdkn2a relative expression
Cdkn1a relative expression
IL-1β relative expression
IL-6 relative expression
PBS
EN4
CRE
CRE+EN4
C
Neutrophils (x10⁴)
Macrophages (x10⁴)
Lymphocytes (x10⁴)
